# Ketogenic interventions enhance REM sleep in females and support memory in aged rats

**DOI:** 10.3389/fnagi.2026.1797686

**Published:** 2026-04-10

**Authors:** Arianna R. S. Lark, Fayaz A. Mir, Naqiya Ambareen, Fangyun Tian, Abigail Hardy Carpenter, Eliza A. Crowley, Bryton A. Toro, Channing E. Syme, Sema Aygar, Ahil Thendral, Kathryn Smith, Christian G. White, Mohammed S. Mahamdeh, Jinyoung Choi, Christa J. Nehs

**Affiliations:** 1 Mass General Brigham Department of Anesthesiology, Massachusetts General Hospital and Harvard Medical School, Boston, MA, United States; 2 Division of Sleep Medicine, Harvard Medical School, Boston, MA, United States; 3 Cardiovascular Research Center, Cardiology Division, Department of Medicine, Massachusetts General Hospital and Harvard Medical School, Boston, MA, United States; 4 Mass General Brigham Center of Excellence for Molecular Imaging, Boston, MA, United States

**Keywords:** aging, cognition, inflammation, ketogenic diet, metabolism, neuroenergetics, oxidative stress, sex differences

## Abstract

**Introduction:**

Sleep disruption and metabolic decline are key contributors to cognitive aging and dementia risk. While cerebral glucose utilization declines with age, ketone metabolism remains relatively preserved, suggesting that ketogenic interventions may enhance brain energetics, sleep quality, and cognition in older individuals. Therefore, we investigated the effects of a ketogenic diet (KD) and *β*-hydroxybutyrate ester (KE) supplementation on sleep–wake architecture and novel spatial memory following sleep deprivation in aged rats.

**Methods:**

Forty four aged (22–25-month-old) Fischer-344 rats were implanted with frontoparietal EEG and nuchal EMG electrodes for wireless sleep recordings. Following baseline assessment, rats received either KE gavage (2.5 g/kg) or water control or were fed a KD versus control diet for 2 months. Sleep stages were scored using AccuSleep, cognition was assessed using the novel place preference task, and postmortem immunohistochemistry was performed to assess oxidative stress and inflammation.

**Results:**

Both KE (*p* = 0.0152) and KD (*p* = 0.0385) significantly increased REM sleep in aged female rats, with KE enhancing REM during the light phase and KD during the dark phase. Whereas, KE decreased REM sleep in males during the light phase (*p* = 0.00165). KD increased the number of REM sleep bouts without altering bout duration in females, consistent with enhanced REM sleep initiation rather than maintenance. EEG spectral analysis revealed no differences in NREM delta power or REM theta power in KD females, suggesting preserved microarchitecture. Sleep deprivation did not change spatial memory in our rats but KD treated rats showed improved novel place recognition in both females and males (*p* < 0.001). Immunohistochemistry revealed reduced lipid peroxidation (4-HNE) without changes in protein oxidation (3-NT) or NLRP3 expression, while TREM2 was selectively reduced in KD females. This pattern is consistent with altered microglial lipid handling rather than broad suppression of inflammatory signaling. In contrast, aged male rats showed no significant changes in sleep architecture or molecular markers.

**Discussion:**

Together, these findings demonstrate that ketone-based interventions selectively enhance REM sleep, reduce lipid-associated oxidative stress in aged female rats and improve novelty preference memory in both sexes. These data highlight a sex-dependent relationship between metabolic flexibility, REM sleep regulation, and cognitive aging, supporting ketone-based strategies as targeted interventions for promoting healthy brain aging.

## 1 Introduction

Aging is accompanied by profound changes in metabolism and brain function that together contribute to neurodegeneration and cognitive decline. Among the most prominent and clinically relevant changes are disruptions in sleep architecture and circadian regulation, including increased sleep fragmentation, reduced sleep efficiency, and phase advances of the sleep–wake cycle (Adam et al., 2006; Backhaus et al., 2007; Mander et al., 2017; Zalai et al., 2017). These alterations arise from converging mechanisms including changes in neurotransmitter signaling (Cleeland et al., 2019), circadian clock dysregulation (Acosta-Rodriguez et al., 2021), and impaired metabolic homeostasis (Amorim et al., 2022). Importantly, sleep disruption in aging is not merely an epiphenomenon but actively contributes to cognitive decline by impairing memory consolidation, synaptic homeostasis, and clearance of neurotoxic metabolites (Wimmer et al., 2013; Xie et al., 2013).

In parallel with sleep disruption, aging is characterized by a progressive decline in cerebral glucose metabolism and mitochondrial efficiency, increasing vulnerability to energetic stress (Yin et al., 2014; Goyal et al., 2017). In contrast to this reduced glucose utilization, the ability of the aged brain to metabolize ketone bodies such as *β*-hydroxybutyrate (BHB) remains relatively preserved (Cunnane et al., 2016). Ketones provide an effective alternative fuel for ATP production (Veech, 2004). Beyond their energetic role, ketones act as signaling molecules that modulate mitochondrial biogenesis, redox balance, inflammation, and synaptic plasticity through epigenetic and metabolic mechanisms (Veech, 2004; Brownlow et al., 2013; Newman and Verdin, 2017; Neth et al., 2020). Long-term ketogenic diets (KD) improve cognitive performance and alter synaptic signaling in aged rats (Hernandez et al., 2018), highlighting the promise of therapeutic metabolic interventions potential to support brain energetics and sleep-related function in aging (Cunnane et al., 2016; Sanderlin et al., 2022).

Emerging evidence suggests that ketone metabolism directly influences sleep–wake regulation. Ketogenic interventions have been proposed to enhance sleep quality, stabilize circadian rhythms, and modulate both non-rapid eye movement (NREM) and rapid eye movement (REM) sleep (O'Hearn, 2021; Westmark et al., 2023; Zhuang et al., 2023). In rodents, central administration of the ketone acetoacetate enhances slow-wave activity during NREM sleep in a dose dependent manner (Chikahisa et al., 2014). Clinical studies report increased REM sleep and improved sleep continuity in pediatric epilepsy (Hallbook et al., 2007) and enhanced sleep quality in adults with multiple sclerosis (Perlman et al., 2024) following ketogenic interventions. In addition, ketogenic interventions have been shown to improve sleep quality in diabetics (Siegmann et al., 2019) and REM sleep after intense exercise (Robberechts et al., 2023). These findings suggest that ketones may influence sleep regulation through metabolic sensing, adenosinergic signaling, or redox-dependent modulation of neuronal excitability.

Importantly, both sleep regulation and metabolic responses exhibit pronounced sex differences across the lifespan (Carrier et al., 2017; Lok et al., 2024). Females display greater sensitivity to REM sleep modulation, distinct mitochondrial redox capacity, and differential sensitivity to metabolic interventions, potentially reflecting interactions between sex hormones, neuronal excitability, and energy metabolism (Gaignard et al., 2015; Mong and Cusmano, 2016; Choi et al., 2021). These differences may be amplified with aging, when metabolic flexibility becomes increasingly important for maintaining sleep quality and cognitive function. Understanding sex-specific mechanisms is therefore essential for developing targeted metabolic therapies for age-related sleep and cognitive disorders.

Despite growing recognition of the interdependence between sleep, metabolism, and cognition, the role of ketone metabolism in regulating sleep–wake dynamics in the aging brain remains poorly understood. Here, we investigated the effects of two complementary ketogenic interventions, exogenous *β*-hydroxybutyrate ester (KE) supplementation or a ketogenic diet (KD) on sleep–wake architecture, EEG spectral features, sleep deprivation, and novel place memory in aged (22–25-month-old) Fischer-344 rats. We further assessed oxidative stress and inflammatory markers to explore potential mechanistic pathways. We hypothesized that increasing ketone availability would enhance sleep quality, reduce oxidative stress and neuroinflammation, and support novel place memory, with possible sex-dependent effects. By integrating electrophysiological, behavioral, and molecular analyses, this study provides novel insight into how metabolic flexibility modulates REM sleep regulation, oxidative stress, and memory in the aging brain.

## 2 Materials and methods

### 2.1 Animal care and use

**Figure 1:**
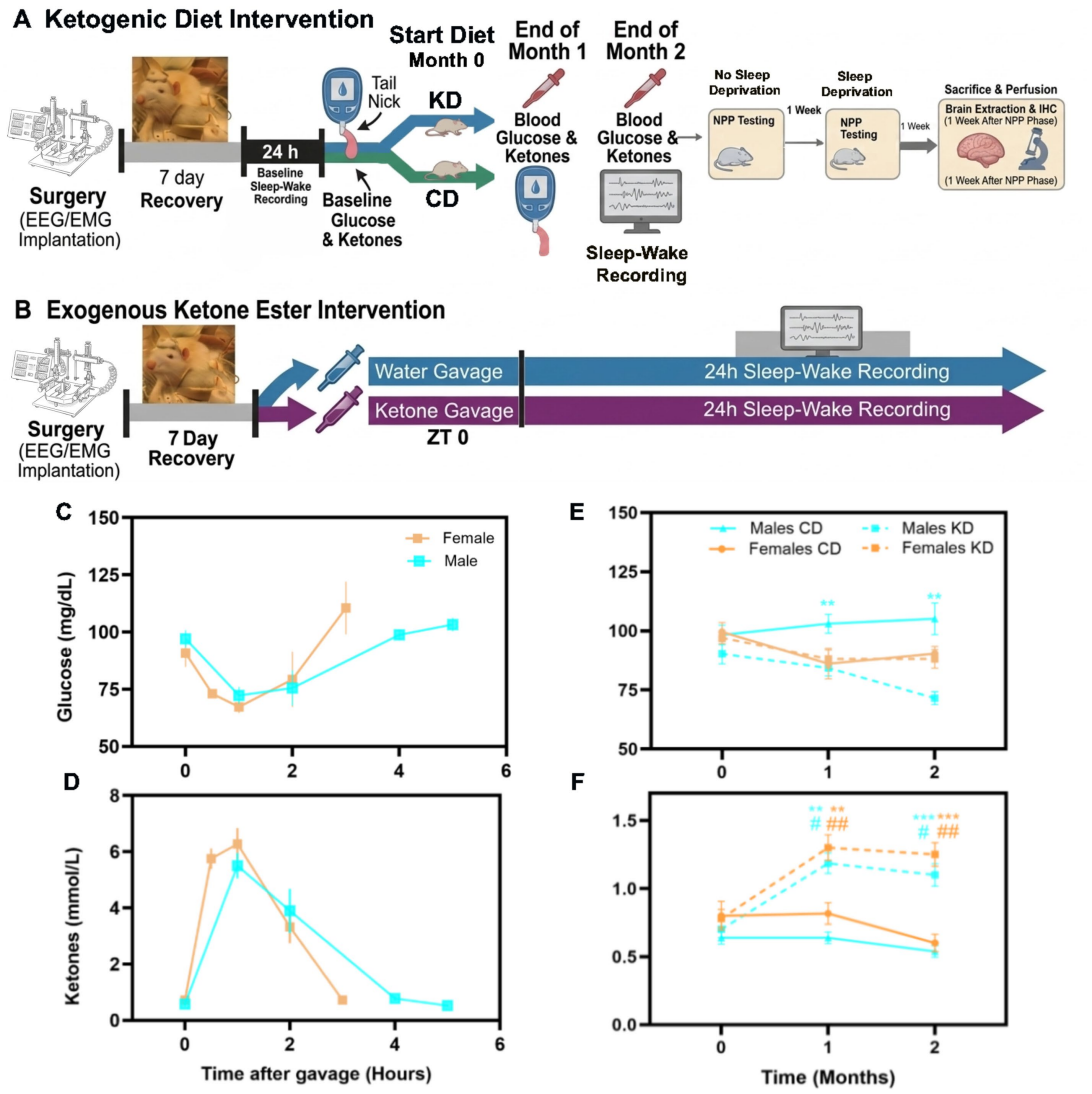
Experimental timeline and metabolic effects of ketone ester and diet interventions in aged male and female rats. **(A,B)** Schematic timelines for dietary **(A)** and oral ketone ester gavage **(B)** interventions. Animals were implanted with EEG/EMG electrodes and allowed 7 days of recovery prior to baseline recordings and blood sampling. In the diet paradigm **(A)**, rats were fed either a control diet (CD) or a ketogenic diet (KD) for 2 months, with blood glucose and ketone measurements taken at baseline, 1 month, and 2 months. In the gavage paradigm **(B)**, rats received oral ketone ester or water at ZT 0, followed by **(C,D)** blood glucose (C) and ketone (D) sampling over the following 3-5 hours post-gavage. **(C,D)** Blood glucose **(C)** and ketone **(D)** concentrations were measured at 0, 2, 4, and 6 h following oral ketone ester administration in aged male and female rats. Ketone ester elevated circulating ketones and transiently reduced glucose levels, with sex-specific dynamics. **(E,F)** Longitudinal blood glucose **(E)** and ketone **(F)** concentrations in male (blue) and female (orange) rats maintained on CD or KD for 2 months. KD significantly elevated circulating ketones in both sexes, with a more pronounced effect in females. Glucose levels were reduced in KD-fed males but remained stable in females. Statistical significance is indicated as *p < 0.05, **p < 0.01, ***p < 0.001, #*p* < 0.05, ##*p* < 0.01, ###*p* < 0.001 (* means comparison across diet groups, #means comparison within the same diet group across time points).

Forty-four, 22–25-month-old Fischer-344 rats (NIA’s old animal colony at Charles River Laboratories, Wilmington, MA) were used in the study. All rats were kept on a 12:12 h (7 a.m.,7 p.m.) light–dark cycle in a temperature and humidity-controlled AAALAC-accredited facility and were housed in pairs until undergoing surgical procedures. Rats were randomly assigned to a ketone gavage group or two dietary groups, a control diet (CD) (Research Diets D19082304i, 10% fat, 10% protein, 80% carbohydrate) or a KD (Research Diets D23072803i, 90% fat (primarily cocoa butter), 10% protein, 0.1% carbohydrate) for 2 months (changed twice weekly). Animals receiving the ketone gavage were fed the CD and received equal volume of 2.5 g/kg of the Oxford ketone monoester (R)-3-hydroxybutyl (R)-3-hydroxybutyrate (DeltaG Tactical, D-BHB which is converted to BHB in the liver) or water by oral gavage administered to the rats between 7:00 a.m. and 7:15 a.m. at the start of the light cycle. Blood glucose and ketone levels were measured at 1, 2, 4 and 5 h post gavage in males and 0.5, 1, 2 and 3 h post gavage in females using a blood glucose and ketone meter (Precision Xtra Blood Glucose & Ketone Monitoring System). Initial pharmacokinetic measurements were performed in males to characterize the temporal profile of circulating ketone levels following ketone ester administration. Based on the rapid early increase observed, additional early time points were included in the female cohort to provide finer temporal resolution during the first hour after administration. Despite the slightly different sampling intervals, both datasets capture the full rise and return toward baseline ketone levels following ketone ester administration. For the dietary group, blood glucose and ketone levels were measured at 0, 1, and 2 months after switching diets (Figure 1). Body weight did not differ between groups (females CD 247.2 ± 23 grams vs. KD 252.85 ± 23.5 grams: males CD 410.86 ± 37 grams vs. KD 397.44 ± 30.5 grams *t*-test *p* > 0.05). Age-related morbidity and mortality, including tumors, bumblefoot, and other causes associated with advanced age, resulted in some animals not completing the study and being excluded from the final analyses. Nine male rats died (4 from bumblefoot, 2 from tumors, 1 from a tail lesion, and 2 unknown cause of death) and 7 female rats were excluded (but only 2 from death due to unknown causes, 3 displayed epileptiform like activity in the EEG, and 2 where the EEG signal quality was poor). All procedures involving animals were approved by the MGH institutional animal care and use committee (Protocol #: 2019N000143 approved Aug 4, 2019) and followed ARRIVE guidelines 2.0 published by NC3Rs in 2020.

### 2.2 Surgical implantation of electroencephalogram and electromyogram electrodes

For surgical implantation of EEG and EMG electrodes, anesthesia was induced with 3% isoflurane in 100% oxygen and maintained at 1.5% and the scalp hair was shaved off with a hair trimmer to expose the skull. Rats were then secured in a stereotaxic frame (David Kopf Instruments, Tujunga, CA). A heating pad maintained the animal’s body temperature at 37 °C throughout the surgery. EEG electrodes (0.005-inch stainless steel, A-M Systems, Sequim, WA) were placed over the prefrontal cortex (AP: +1.5 mm, ML: ±1.5 mm) and parietal cortex (AP: −1.5 mm, ML: ±1.5 mm) relative to bregma and a 2EEG/1EMG prefabricated head mounts (8200-K9-SL2 EEG/1-EMG wireless transmitter, Pinnacle Inc., United States) with platinum iridium or stainless steel leads were implanted connecting all the EEG screws to their respective sites. Additionally, 5 stainless steel anchor screws were implanted to support the headmount and transmitter head cap (1.59 mm diameter, 3.2 mm long, Stoelting Co. Wood Dale, IL). The EMG electrodes pre-attached with the headmount were placed into the nuchal muscle. A headmount cap (Pinnacle Inc., United States) was placed atop of the headmount female connector and to accommodate the Bluetooth transmitter during the EEG recording. Finally, the headmount, EEG and EMG wires as well as cap were together secured with acrylic dental cement to the skull (Teets Dental Cement, A-M Systems, Sequim, WA). Surgeries lasted 2–3 h. For post-operative care, rats were given ketoprofen (5 mg/kg) and 10 mL of normal saline every 24 h as needed until fully recovered. Rats were given at least 7 days to recover before experiments and were housed individually for the rest of the study. A baseline sleep recording was performed to acclimate animals to the recording room (8021).

### 2.3 Polysomnographic recording and sleep scoring

Polysomnographic recordings were conducted using a Pinnacle wireless telemetry system (Pinnacle Inc., United States) equipped with 2 EEG and 1 EMG channels for continuous 24-h monitoring. Wireless sleep–wake recording prevents restraint stress often faced during the traditional tethered systems. Data acquisition was carried out using Sirenia Acquisition software with a bandwidth upper cut off at 40 Hz and lower cut off at 0.1 HZ as well as EMG signal was bandwidth filtered at 10–90 Hz (Pinnacle Inc., United States). Sleep recordings were saved offline for analysis. For sleep–wake staging, EEG files were subsequently exported in European Data Format (EDF) format and converted to MATLAB (.mat) format for analysis in MATLAB. Sleep was scored in 10 s epochs in AccuSleep in MATLAB. AccuSleep requires user training of sleep–wake states and then automatically classifies sleep–wake stages into wake, NREM and REM sleep. Vigilant states such as wake (marked by low amplitude high frequency with prominent EMG activity), NREM sleep (0.5–4 Hz, with lower EMG activity and high amplitude low frequency), and REM sleep (5–8 Hz with high frequency low amplitude waves with muscle atonia) stages. This automated scoring approach provided objective and consistent classification of sleep architecture across all experimental subjects throughout the entire 24-h recording period and was further verified by eye before finalization to a MATLAB label file. MATLAB label files were then assessed for percentage of wake, NREM and REM sleep, number of bouts, and bout length using MATLAB scripts. Further analysis of state dependent oscillation power was assessed using scripts assessing the EEG EDF and the label file and lab scripts and EEGLAB. All data were exported to Microsoft Excel and statistical assessment and graphing was completed using GraphPad Prism version 10 or MATLAB.

### 2.4 Sleep deprivation with gentle handling

Acute sleep deprivation was used as a physiological challenge to assess sleep homeostasis and resilience to sleep disruption in aged animals. Sleep deprivation for 6 h was carried out by the gentle handling method, a widely accepted technique for preventing sleep in rodents while minimizing stress. During the sleep deprivation, rats were continuously monitored and EEG/EMG were simultaneously recorded by a wireless Pinnacle system. Rats were prevented from falling asleep using gentle handling and gently stroking the animals whenever behavioral or EEG signs of sleep onset were detected. During the sleep deprivation food and water was given ad-libitum to avoid hunger and thirst associated stress in animals. This method allows for sleep deprivation while preserving normal locomotor activity and minimizing the activation of stress-response pathways that could confound experimental results.

### 2.5 Novel placement preference test

Cognitive effects of the dietary intervention on novel place memory were assessed using the novel place preference test. On the training day, two identical objects were placed in the corners of the open field chamber (50 × 50 × 35 cm, width x length x height) and the animal was placed in the arena and allowed to explore each object for 10 min. During the test phase, 24 h after the training trial, one of the objects was moved to a new location and the animal was allowed to explore both objects for 5 min. An animal’s exploration time was recorded via a video camera mounted above the chamber and processed using ANY-maze software. The time the animal spent exploring the object at a novel place in relation to the object that did not move (in a 30 mm oval around an object) was manually scored using a timer. The ratio of time spent with the familiar object to that of time spent near the novel location was calculated using the discrimination index (DI). Discrimination indices were calculated as $\left(T_{\text{novel}}-T_{\text{familiar}})/(T_{\text{novel}}+T_{\text{familiar}}\right)$ and linearly transformed to a 0–1 scale, where 0.5 represents equal exploration of novel and familiar locations and 1 indicates the novel location and 0 indicates the familiar location. Each dietary group and sleep deprivation condition was compared to each other using a two-way repeated-measure ANOVA. Due to advanced age–related variability in spontaneous exploration, animals that failed to meet a predefined minimum exploration criterion (total object exploration <5 s) were excluded from behavioral analysis. Female and male exclusion rates were similar at 19% (females 7 of 36 trials: males 5 of 26 trials). This exclusion was applied to ensure that performance reflected memory-based exploration rather than age-related reductions in locomotion, motivation, or task engagement, which are common in very old rodents.

### 2.6 Immunohistochemistry

Rats were anesthetized with 3% isoflurane and transcardially perfused with 1x PBS followed by 10% formalin for brain tissue fixation. Brains were extracted, and 50 μm sections were cut using a Leica VT1000 S vibrating blade vibratome. For histology examination, brain sections were permeabilized using phosphate buffered saline with triton (PBST) (0.1% Triton X-100 in PBS) for 30 min at room temperature, then incubated in a blocking solution (4% normal goat serum in PBS) for 2 hours at room temperature before overnight incubation with the primary antibody at 4 °C. Following incubation in the primary antibodies, the brain sections were then washed with PBS three times before applying the secondary antibodies to incubate for 1 hour at room temperature. Then, the brain slices were washed and mounted on slides using ProLong Glass Antifade Mountant. Slides were imaged using NIKON W1-SoRa spinning disk microscope in the Mass General Brigham Center of Excellence for Molecular Imaging Core.

The following primary antibodies were used: rabbit anti- 4-hydroxynonenal (4HNE) (1:500; Bioss bs-6313R), rabbit anti- 3-nitrotyrosine (3-NT) (1:200; Bioss BS-8551R), rabbit anti- triggering receptor expressed on myeloid cells 2 (TREM2) (1:200; Bioss BS-2723R), rabbit anti- NLR family pyrin domain containing 3 (NLRP3) (1:100; NBP2-12446), mouse anti-beta- III tubulin (1:400; Abcam AB78078), rat anti- ionized calcium-binding adaptor molecule 1 (IBA) (1:1000; Abcam AB283346), chicken anti- glial fibrillary acidic protein (GFAP) (1:1000; Abcam AB4674). The following secondary antibodies were used: goat anti-mouse-594 (1:1000; Invitrogen A11005), goat anti-rat-647 (1:1000; Invitrogen A21247), goat anti-chicken-405 (1:1000; Invitrogen A48260), goat anti-rabbit-488 (1:1000; Abcam AB150077).

### 2.7 Data analysis

All statistical analyses were performed using MATLAB and/or GraphPad Prism version 10.

#### 2.7.1 Immunohistochemistry analysis

A hybrid Ilastik-QuPath method was used for the batch quantification of oxidative stress and inflammatory marker intensity in each cell type. Immunofluorescence images were preprocessed in NIH-ImageJ software to adjust the background and contrast, then cropped to the CA1 region of the hippocampus. For each marker, images were then placed in a single folder and labeled using a consistent naming convention as Image01, Image02, and so on for downstream analysis. The order of the channels was consistent for all the cropped images. A few representative images were imported into Ilastik for pixel classification and cell segmentation. Under feature selection, all the default Ilastik features like Raw, Gaussian smoothing, etc. were selected for training and pixels were labeled as neurons, microglia, astrocytes or background. The live update feature of Ilastik was used to verify cell segmentation and prediction of each cell type. After validation, batch processing was run to generate probability maps for all images, which were saved to a new directory. The cropped images and their corresponding probability maps were overlaid and exported as a merged composite image to a third output folder using a custom Groovy based Fiji macro. Composite images were imported into QuPath as a new project. “Create full image annotation” was selected for analysis of the whole image to analyze the entire field of view. Parameters for cell detection were selected carefully for correct identification of neurons, microglia and astrocytes. Thresholds were optimized separately for each cell type. Intensity settings were adjusted to fine-tune the sensitivity of detection based on probability maps. Oxidative stress markers, 4HNE and 3NT, and neuroinflammation markers, TREM2 and NLRP3 signals were labeled using the goat anti-rabbit-488 antibody in the FITC channel (green). After finalizing the specific parameters for cell detection, a customized script was run for batch processing to quantify intensity of the FITC channel in each of the cell types independently. The output included two directories, one with the .csv files, where the last column provided the mean FITC intensity in the neurons, microglia and astrocytes, respectively. A second output folder contained the images of all the detections for each of the images run in that QuPath project. The images were quality checked and false detections were eliminated to ensure that cell detection is accurate. The poor detections were eliminated using a custom code in MATLAB and the cleaned values were then averaged for each image to give a final output containing the image number and mean FITC intensity in individual cell types.

Immunofluorescence quantification was performed at the field-of-view level across multiple hippocampal sections per animal (one field of view per section, three sections per rat, ~100–200 cells quantified per slice for each cell type). These measurements represent observations within each animal and allow sampling of cellular heterogeneity across hippocampal regions. The biological unit of replication in the study is the animal, while field- and cell-level measurements provide higher-resolution estimates of signal intensity within each animal, consistent with prior hippocampal imaging studies (Jin et al., 2020; Zhang et al., 2024). Because oxidative stress and inflammatory signaling in the aged hippocampus can exhibit substantial cellular and regional variability, quantification at the field-of-view level enables sampling of this heterogeneity while accounting for clustering of measurements within animals. Accordingly, signal intensity and cell counts were quantified per image/field, consistent with common practice in quantitative hippocampal immunohistochemical analyses. To account for the hierarchical structure of the data, statistical analysis was performed using a mixed-effects two-way ANOVA (sex × diet) with animal included as a random factor to account for nested sampling of fields of view within animals. Data were organized in GraphPad Prism (v10.6.1) using nested data tables so that measurements obtained from the same animal were treated as related observations rather than independent replicates, thereby avoiding pseudoreplication. *Post hoc* comparisons were performed using Tukey’s multiple comparisons test.

To quantify the overall intensity of the 4HNE and the number of 4HNE puncta, cropped images were run for batch processing to quantify the integrated density of the third channel. The settings for batch analysis were determined based on a previous report (Aron et al., 2023). A custom code was run in Fiji macro to convert composite images to 8-bit, split channels and apply all the settings to the FITC channel, subtract background (the rolling ball radius set at 25), denoising by applying the Gaussian blur filter (radius set at 1 pixel), thresholding was set at a specific range for 4HNE for particle analysis to calculate puncta number. This macro was applied to the entire folder of cropped images. The output yielded an excel sheet with the image number, integrated density of 4HNE, number of 4HNE puncta, area of the field in μm^2^. Graphs were plotted as integrated density per mm^2^ and number of puncta/mm^2^.

TREM2 expression in the brain is largely restricted to microglia (Ulland et al., 2017). Accordingly, TREM2 quantification focused primarily on microglial signal. We also observed TREM2 immunoreactivity associated with neuronal markers, which likely reflects binding or uptake of soluble TREM2 shed from microglia rather than neuronal expression of the receptor (Henjum et al., 2016; Zhang et al., 2023). In contrast, NLRP3 inflammasome components have been reported in multiple CNS cell types, including microglia, astrocytes, and neurons, particularly under aging or inflammatory conditions (Freeman et al., 2017; Voet et al., 2019; Cihankaya et al., 2024). Accordingly, NLRP3 expression was evaluated across these cell populations in the aged rat hippocampus. The same workflow was followed for TREM2, NLRP3 and 3NT except that only the overall integrated density of these markers was tabulated as it showed a diffused rather than punctate staining pattern. The output yielded an excel file containing integrated density and analyzed area in μm^2^. The integrated density per mm^2^ was then plotted using the GraphPad Prism software and compared across groups for statistical analysis.

#### 2.7.2 EEG spectral analysis

To quantify spectral dynamics across sleep stages, time-frequency analysis of EEG data was carried out using multitaper spectral estimation techniques with a 10-s moving window, time half-bandwidth product of 5 and 9 discrete prolate spheroidal sequences as tapers in MATLAB using the script. This method provides a vigorous spectral analysis by reducing variance and spectral leakage, highly desirable for analyzing nonstationary EEG signals. Preprocessed EEG data was first scored using AccuSleep and then the label file in MATLAB format, as well as its parent EDF EEG file, were loaded into the MATLAB-EEGLab ecosystem for spectral analysis. The signals were then analyzed by a 10-s moving window and delta power (0.5–4 Hz) was extracted specifically from all NREM sleep episodes across 24 h of sleep recording. NREM delta power for each animal was averaged to obtain mean delta power per animal. Similarly, using the modified MATLAB code for each frequency such as delta (0.5–4 Hz), theta (5–8 Hz), and their ratios (delta/theta), changes across sleep–wake states were computed and plotted as time-frequency graphs. Spectral density across groups (diet versus time) was statistically compared using 2-Way ANOVA and percent change from baseline (each animal served its own baseline for the effects of diet on time-dependent frequency changes across sleep–wake) were compared using 2-way ANOVA test, respectively. Figures and time-frequency data were then plotted in GraphPad Prism version 10.

## 3 Results

### 3.1 Exogenous ketone esters and ketogenic diet effect on blood ketone and glucose levels

Figures 1A,B illustrate the experiment timeline for the exogenous KE and KD interventions. Both approaches robustly increased circulating ketone levels, with distinct temporal dynamics and sex-specific effects on glucose metabolism.

Acute effects of KE gavage: Following oral KE gavage, blood ketone and glucose levels changed rapidly in both sexes (Figures 1C,D). Repeated-measures ANOVA revealed a significant decline in blood glucose levels after KE administration in males (*F*(4, 12) = 9.80, *p* < 0.05) and females (*F*(4, 12) = 6.91, *p* < 0.05). In parallel, blood ketone levels rose significantly following KE gavage in both males (*F*(4, 12) = 26.30, *p* < 0.001) and females (*F*(4, 12) = 14.92, *p* < 0.001). Relative to baseline (time 0), blood ketone levels in males were significantly elevated at 1 h (*p* = 0.0012) and 2 h (*p* = 0.0052) post-gavage. In females, ketone levels increased earlier and remained elevated at 0.5 h (*p* = 0.0016), 1 h (*p* = 0.0008), and 2 h (*p* = 0.0052) (Figure 1D). Correspondingly, blood glucose levels declined significantly in males at 1 h (*p* = 0.0132) and 2 h (*p* = 0.0100) post-gavage, while females showed significant glucose reductions at 0.5 h (*p* = 0.048) and 1 h (*p* = 0.0344) (Figure 1C). These results confirm that exogenous ketone ester administration produced a rapid increase in circulating ketone levels accompanied by a transient reduction in blood glucose in both sexes.

Chronic effects of KD: Long-term KD feeding also produced sustained elevations in circulating ketones (Figure 1F). Two-way ANOVA followed by Sidak’s *post hoc* tests revealed significantly higher blood ketone levels in KD-fed males (*F*(1, 13) = 47.13, *p* < 0.0001) and females (*F*(1, 16) = 33.43, *p* < 0.0001) compared with CD animals (Figure 1F). In males, ketone levels were elevated after both 1 month (*p* < 0.0001) and 2 months (*p* = 0.0002) of KD feeding. Similarly, females showed significantly increased ketone levels at 1 month (*p* = 0.0004) and 2 months (*p* = 0.0001). KD feeding also differentially affected blood glucose levels by sex. KD males exhibited a significant overall reduction in blood glucose compared with CD males (*F*(1, 13) = 24.38, *p* < 0.001), with glucose levels significantly lower at both 1 month (*p* = 0.0032) and 2 months (*p* = 0.0011) (Figure 1E). In contrast, blood glucose levels did not differ between KD and CD females (*F*(1, 16) = 0.1438, *p* = 0.7095), and no significant differences were observed at either time point (*p* > 0.05) (Figure 1E).

Taken together, these results confirm that both acute exogenous KE supplementation and chronic KD feeding effectively elevate circulating ketone levels. However, the accompanying effects on glucose metabolism differ by sex, with males showing more sustained glucose reductions with KD.

### 3.2 Exogenous ketones enhance REM sleep in females while reducing REM sleep in males

**Figure 2:**
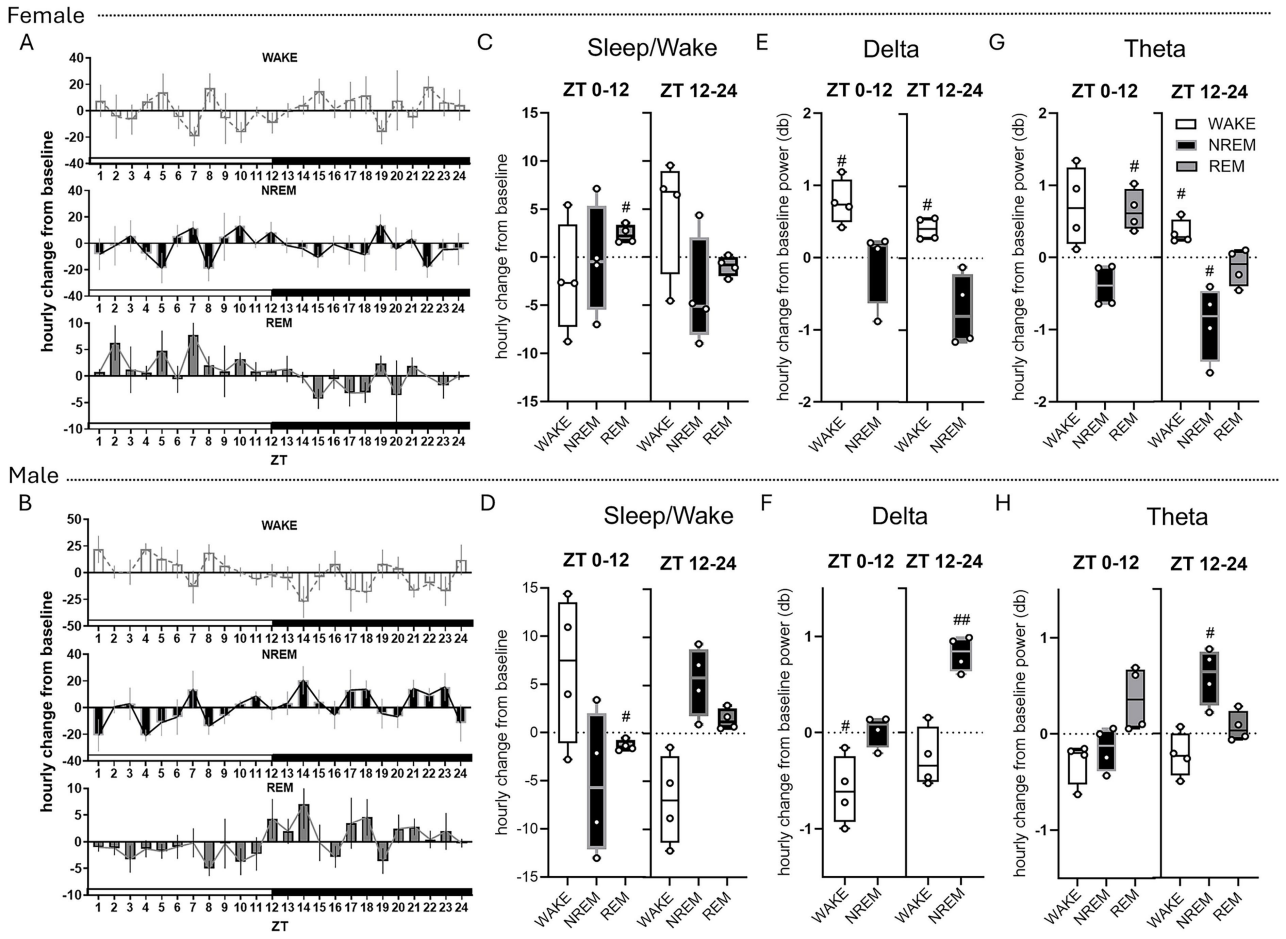
Exogenous ketones modulate sleep and wake features differentially in males and females. Hourly change from baseline in wake, NREM, and REM in females (*n* = 4) **(A)** and males (*n* = 4) **(B)** over the 24 h after ketone ester gavage. Average change in wake, NREM, REM from baseline during ZT 0–12 and ZT 12–24 in females **(C)** and males **(D)**. Average change from baseline in delta during wake and NREM from ZT 0–12 and ZT 12–24 in females **(E)** and males **(F)**. Average change from baseline in theta during wake, NREM, REM from ZT 0–12 and ZT 12–24 in females **(G)** and males **(H)**. #*p* < 0.05, ##*p* < 0.005 assessed via one-sample *t*-test.

Exogenous KE supplementation produced marked sex-dependent effects on sleep architecture, particularly REM sleep. In female rats, KE significantly increased time spent in REM sleep compared with baseline water gavage during the light phase (Zeitgeber Time (ZT) 0–12; *p* = 0.0152, one-sample *t*-test; Figures 2A,C). No significant changes were observed in wake or NREM sleep in females across either light or dark phases (Figures 2A,C). In contrast, male rats exhibited a reduction in REM sleep following KE supplementation during the light phase (ZT 0–12; *p* = 0.00165, one-sample *t*-test; Figures 2B,D). During the subsequent dark phase (ZT 12–24), males showed a trend toward reduced wakefulness (*p* = 0.0586) and increased NREM sleep (*p* = 0.0567; Figures 2B,D), suggesting a redistribution of vigilance states across the circadian cycle. In both sexes, KE supplementation did not alter REM sleep duration during the dark phase (ZT 12–24).

To further assess the qualitative features of sleep and wake states, we analyzed EEG spectral power during wake, NREM, and REM sleep. In female rats, KE supplementation increased delta power during wakefulness relative to baseline during both the light (*p* = 0.0169) and dark (*p* = 0.0132) phases (Figure 2E). In males, delta power during wakefulness decreased during the light phase (ZT 0–12; *p* = 0.0438; Figure 2F) but increased during NREM sleep in the dark phase (ZT 12–24; *p* = 0.0028; Figure 2F). Theta power exhibited additional sex- and state-dependent modulation. In females, theta power increased during REM sleep in the light phase (ZT 0–12; *p* = 0.020; Figure 2G), accompanied by increased theta during wakefulness in the dark phase (*p* = 0.0257) and decreased theta during NREM sleep in the dark phase (*p* = 0.0386). In males, theta power increased during NREM sleep in the dark phase (ZT 12–24; *p* = 0.0271; Figure 2H). Together, these findings demonstrate that exogenous ketone supplementation differentially modulates REM sleep, vigilance state distribution, and oscillatory activity in females and males, revealing a pronounced sex-dependent effect of ketone metabolism on sleep architecture and EEG dynamics.

### 3.3 The ketogenic diet increased REM sleep in females by increasing REM episode number during the dark phase, with no effect on sleep in males

**Figure 3:**
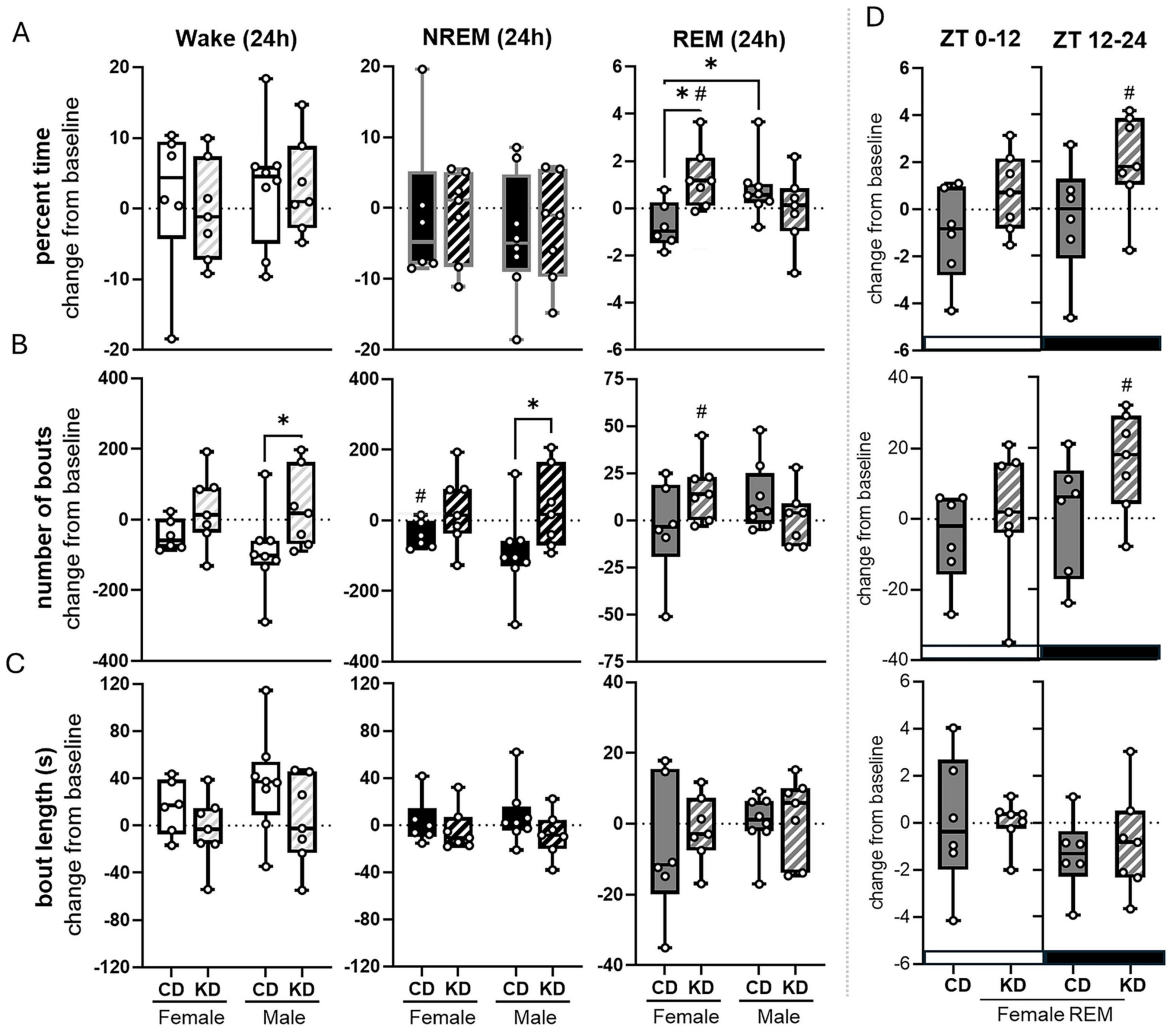
The ketogenic diet increased REM sleep compared to baseline in aged female but not male rats. Data show the change from baseline at month 0 compared to after 2 months of dietary (ketogenic or control) intervention. Change from baseline wake, NREM, and REM in females and males **(A)**. Number of wake, NREM, and REM bouts in females and males **(B)**. Average length of wake, NREM, and REM bouts females and males **(C)**. Female REM changes were broken down further by light vs. dark periods **(D)**. Change from baseline was assessed via one-sample *t*-test. #*p* < 0.05. Change between groups was assessed via two-way ANOVA *p < 0.05 Female Control *n* = 6, Female Ketone *n* = 7, Male Control *n* = 8, Male Ketone *n* = 7.

Animals maintained on the KD for two months exhibited sex-specific differences in sleep–wake architecture relative to baseline. Although there were no main effects of diet or sex on the change from baseline in overall wakefulness or NREM sleep, we identified a significant sex × diet interaction for the change in percent time spent in REM sleep (*p* = 0.0078, two-way ANOVA; Figure 3A). In female rats, the KD significantly increased total REM sleep compared with both their baseline (*p* = 0.0385, one-sample *t*-test) and CD females (*p* = 0.0106, Fisher’s LSD; Figure 3A). To determine the mechanism underlying increased REM sleep in females, we examined REM sleep episode dynamics. Analysis of REM bout number revealed a sex × diet interaction (*p* = 0.0432) and a trend toward increased REM episodes in KD females compared with CD females (*p* = 0.0673). Relative to baseline, KD females exhibited a significant increase in REM bout number (*p* = 0.0382; one-sample *t*-test; Figure 3B), indicating that the increase in REM sleep was driven primarily by enhanced REM episode initiation rather than prolonged REM duration. Further analysis revealed that the REM-promoting effect of KD in females was phase-specific, occurring exclusively during the dark phase (ZT 12–24). During this period, KD females exhibited a significant increase in total REM sleep compared with baseline (*p* = 0.0424; Figure 3D), accompanied by a corresponding increase in REM bout number (*p* = 0.0395; Figure 3D). No significant changes in REM sleep were observed during the light phase.

In contrast, male rats showed no significant changes in wakefulness, NREM sleep, or REM sleep after the two-month dietary intervention in either diet group (Figure 3A). However, analysis of bout structure revealed a main effect of diet on the change from baseline in both wake bout number (*p* = 0.0178; two-way ANOVA; Figure 3B) and NREM sleep bout number (*p* = 0.0173; two-way ANOVA; Figure 3B). CD males showed a reduction in both wake bouts (*p* = 0.0278; Fisher’s LSD) and sleep bouts (*p* = 0.0243; Fisher’s LSD) relative to KD males (Figure 3B). In female CD animals, we observed a modest reduction in NREM sleep bout number (*p* = 0.0440; Figure 3B), with no accompanying changes in wake bouts, total NREM sleep, or NREM bout length (Figures 3A–C). Total sleep time was unchanged for both sexes. Collectively, these findings indicate a sex-specific effect of the KD on REM sleep expression, characterized by increased REM sleep episode number during the active (dark) phase in females, while male sleep architecture remained largely unchanged.

### 3.4 Dietary effects on EEG power spectrum dynamics during sleep and wakefulness

**Figure 4:**
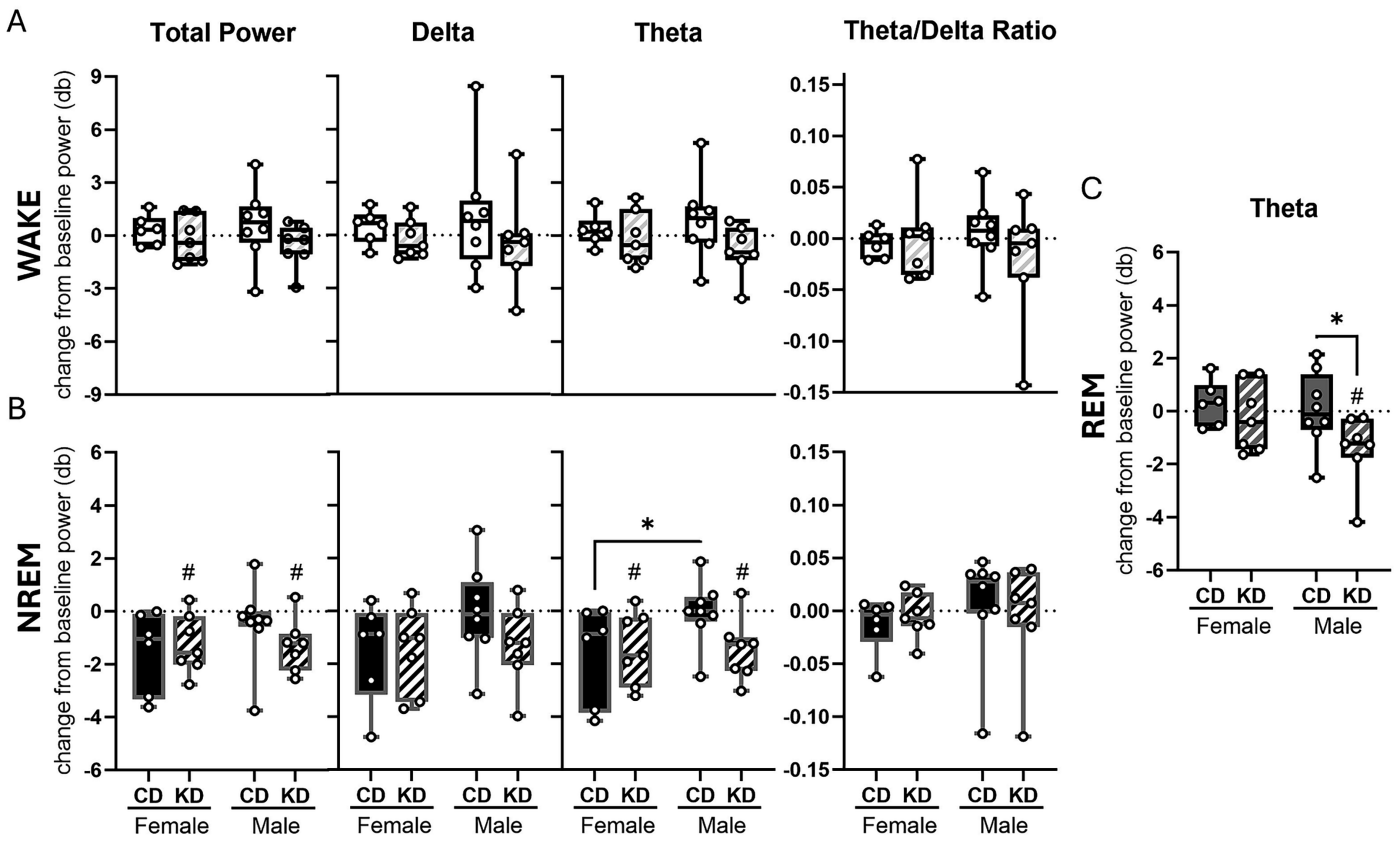
The ketogenic diet reduced the power of theta oscillations and total oscillation power during NREM in both males and females. Change from baseline in total power, delta, theta, and theta delta ratio power at month 0 compared to after 2 months of dietary (ketogenic or control) intervention. Average change in total, delta, theta, and theta delta ratio power during NREM in females and males **(A)**. Average change in total, delta, theta, and theta delta ratio power during wake in females and males **(B)**. Average change in theta power during REM in females and males **(C)**. Change from baseline was assessed via one-sample *t*-test. #*p* < 0.05 Change between groups was assessed via two-way ANOVA. *p < 0.05 female control *n* = 6, female ketone *n* = 7, male control *n* = 8, male ketone *n* = 7.

To assess whether KD altered the depth or quality of sleep and wakefulness, we quantified EEG spectral power in relevant frequency bands, including delta, theta, the theta-to-delta ratio, and total power during wake and NREM sleep, as well as theta power during REM sleep. During wakefulness, KD did not significantly alter delta power, theta power, the theta-to-delta ratio, or total EEG power in either females or males (Figure 4A), indicating no detectable effects of diet on waking EEG dynamics. During NREM sleep, both KD-fed females and males exhibited a significant reduction in total EEG power relative to their respective baselines (females: *p* = 0.0265; males: *p* = 0.0146; one-sample *t*-tests; Figure 4B). Theta power during NREM sleep was also significantly reduced in KD-fed females (*p* = 0.033) and males (*p* = 0.017; Figure 4B) with a trend toward a significant decline in KD males compared to CD males (*p* = 0.0613). CD females showed a similar trend toward reduced total power (*p* = 0.0625) and theta power (*p* = 0.0843) during NREM sleep, although these changes did not reach statistical significance (Figure 4B). NREM theta was significantly different between CD-fed females and males (*p* = 0.0484). During REM sleep, we found a main effect of diet on REM theta (*p* = 0.0488) with a diet-specific effect emerging in males only. KD-fed males showed a significant reduction in theta power during REM sleep relative to baseline (*p* = 0.0297; one-sample *t*-test; Figure 4C) and CD (*p* = 0.0349, Fischer’s LSD; Figure 4C), whereas no significant changes in REM theta power were observed in females (Figure 4C). Together, these findings indicate that the KD selectively modulates EEG spectral characteristics during sleep, with reductions in total and theta power during NREM sleep in both sexes and a male-specific reduction in REM theta power, while leaving waking EEG dynamics unchanged.

### 3.5 Dietary effects on recovery sleep following acute sleep deprivation

**Figure 5:**
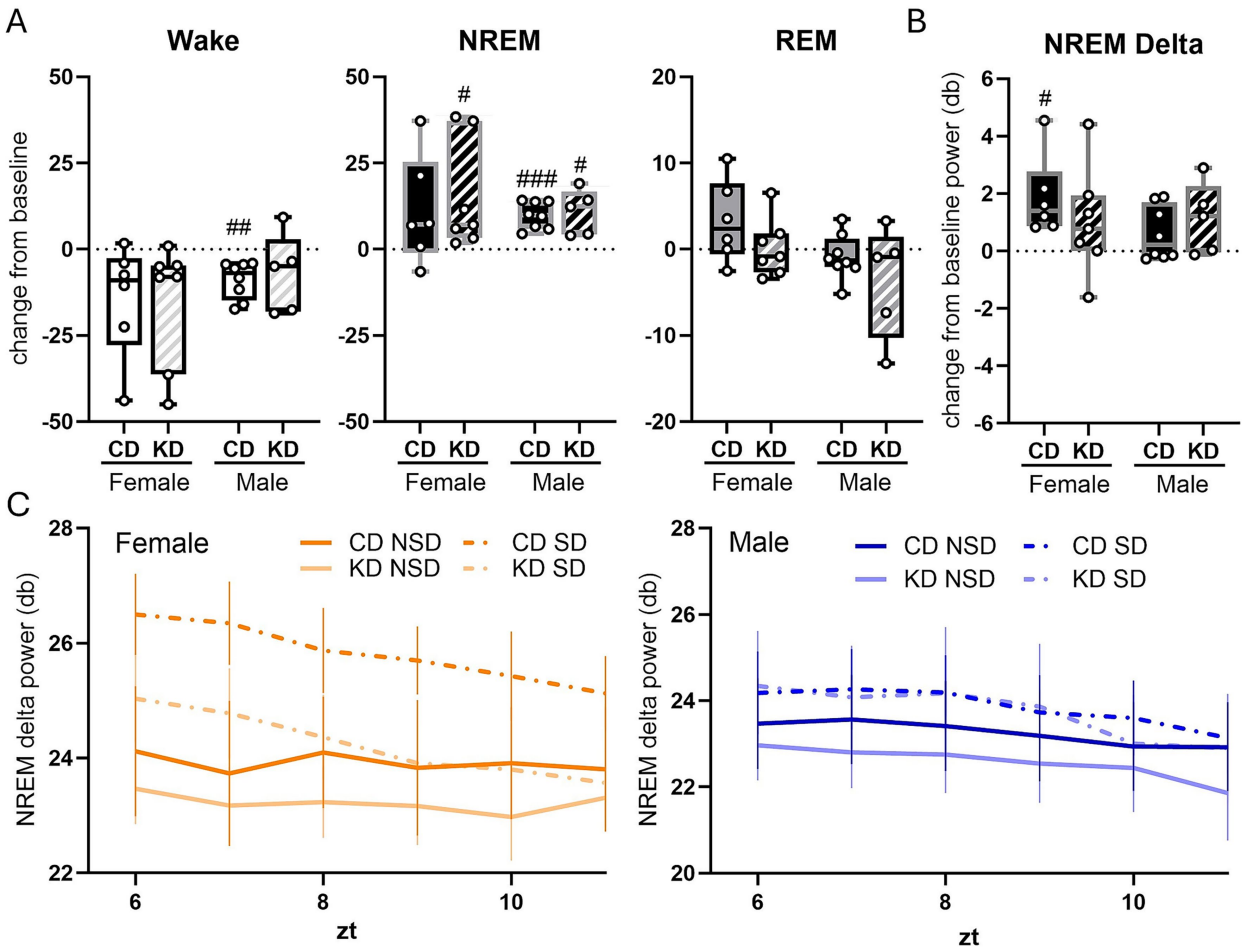
The control and ketogenic diet animals did not exhibit significant differences from each other in the structure of recovery sleep or delta power after 6 h of sleep deprivation. Change from baseline in wake, NREM, and REM during 6 h recovery period after sleep deprivation in female and male **(A)** rats. Average hourly change from delta power during NREM in female and male **(B)** rats. Delta power during NREM from ZT6 to ZT11 in baseline sleep in non-sleep deprived control (control NSD) and ketogenic (ketogenic NSD) or acutely sleep deprived control (control SD) and ketogenic (ketogenic SD) female and male **(C)** rats. Change from baseline was assessed via one-sample *t*-test. Change between groups was assessed via two-way ANOVA. Change in sleep overtime was assessed via mixed effect model followed by Tukey’s *post hoc* analysis #*p* < 0.05 ##*p* < 0.005 ###*p* < 0.0005 female control *n* = 6, female ketone *n* = 7, male control *n* = 8, male ketone *n* = 7.

To determine whether KD alters sleep homeostasis and recovery sleep following acute sleep loss, we examined sleep–wake rebound after 6 h of sleep deprivation. To account for circadian influences and assess changes on an individual basis, hourly sleep–wake values during the recovery period were normalized to each animal’s baseline values at the corresponding ZT under non–sleep-deprived conditions. Recovery sleep was analyzed over the first 6 h following sleep deprivation. In response to sleep deprivation, female rats exhibited a trend toward reduced wakefulness during the recovery period in both CD (CD; *p* = 0.0851) and KD groups (*p* = 0.0651; Figure 5A). KD-fed females showed a significant increase in NREM sleep during recovery (*p* = 0.046; Figure 5A, middle panel), whereas no significant changes in REM sleep were observed in either diet group over the initial 6-h recovery window. In males, sleep–wake rebound differed by diet. CD males exhibited a significant reduction in wakefulness following sleep deprivation (*p* = 0.0022; Figure 5A), whereas KD males did not show a significant decrease in wake time. Both CD and KD males displayed a robust increase in NREM sleep during recovery (CD: *p* = 0.0002; KD: *p* = 0.0203; Figure 5A), with no significant changes in REM sleep in either group.

To assess the intensity of recovery sleep, we examined delta power during NREM sleep across the recovery period and as an average change from baseline. In females, delta power during NREM sleep was significantly elevated immediately following sleep deprivation and declined over time in both CD and KD groups (*p* < 0.0001, ZT6–ZT12; Figure 5C). Only CD females exhibited a significant overall increase in average NREM delta power relative to baseline (*p* = 0.0219; one-sample *t*-test; Figure 5B), whereas KD females did not. In males, KD-fed animals showed a modest but transient elevation in NREM delta power during early recovery that declined over time (*p* = 0.0007, ZT6–ZT12; Figure 5C). Together, these findings indicate that the KD differentially modulates homeostatic sleep responses following acute sleep deprivation, enhancing NREM rebound in females while altering wake suppression and delta power dynamics in males.

### 3.6 The ketogenic diet supports novel spatial memory performance in aged rats

**Figure 6:**
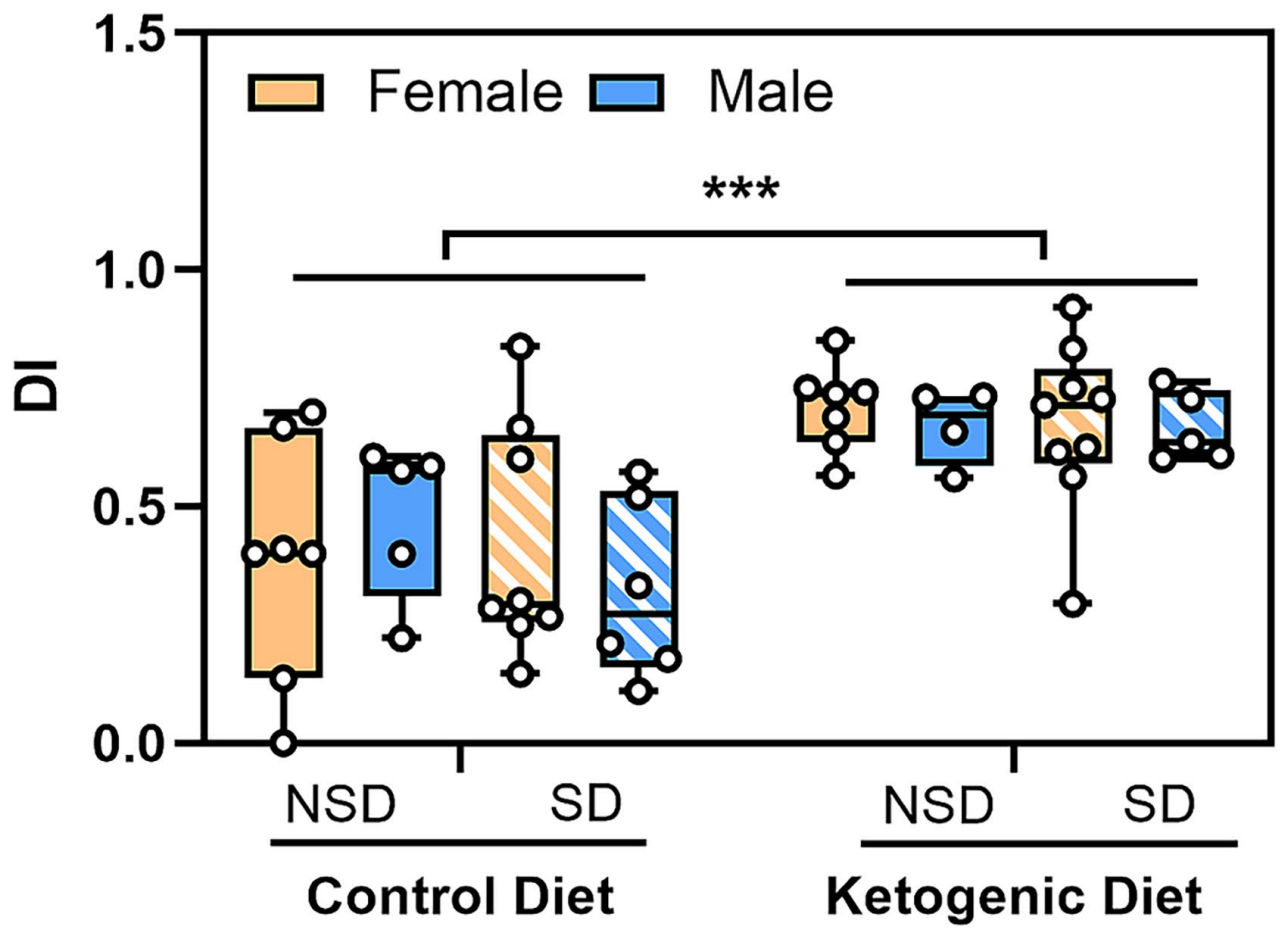
The ketogenic diet improves spatial memory in aged rats. Novel place preference test discrimination index (DI) was calculated in aged female and male rats maintained on control diet (CD) or ketogenic diet (KD) under baseline no sleep deprivation (NSD) and sleep deprivation (SD) conditions. Some old animals had low task participation so a threshold of <5 s of total object exploration time was used to exclude low task engagement animals. Females: CD *n* = 8 originally (NSD: 1 excluded due to low task involvement; SD: none excluded), KD *n* = 10 originally (NSD: 3 excluded due to low task involvement; SD: 1 excluded due to low task involvement). Males: CD *n* = 8 originally (NSD: 3 excluded due to low task involvement; SD: 1 excluded due to low task involvement), KD *n* = 5 originally (NSD: 1 excluded due to low task involvement; SD: non excluded). Box plots display median (center line), interquartile range (box), and minimum/maximum values (whiskers), with individual data points overlaid. Our sleep deprivation protocol did not significantly change memory, however KD rats displayed better memory of the novel place compared to CD fed rats across both sexes suggesting that KD was protective for memory. Three-way between subjects ANOVA followed by Bonferroni post-hoc test revealed significant main effects of diet only in both females and males [*F*(1, 43) = 27.36, *p* < 00.001]. ***p < 0.001. DI values closer to 1.0 indicate greater discrimination for the novel placed object, values closer to 0 indicate greater discrimination for the familiar placed object, while 0.5 indicates no preference between either object location/equal time exploring both objects.

Because aging is associated with a decline in learning and memory, we assessed whether long-term KD feeding supports spatial discrimination in aged rats using the novel place preference task, both under baseline conditions and following sleep deprivation. Discrimination Index (DI) was significantly higher in KD-fed animals compared with CD animals in both sexes (Figure 6) (*F*(1, 43) = 27.36, *p* < 0.001) three-way between subjects ANOVA followed by Bonferroni post-hoc analysis) indicating enhanced preference for the novel location in KD-fed rats. We did not find any sex-dependent significant differences (*F*(1, 43) = 0.085, *p* = 0.773 or sleep-deprivation induced changes in DI scores (*F*(1, 43) = 0.511, *p* = 0.479), which may be due to the animals recovery sleep prior to behavioral testing. Relative to chance performance (DI = 0.5), KD-fed rats exhibited a significant preference for the novel location in both females and males, with and without sleep deprivation (one-sample *t* test vs. 0.5: females KD no sleep deprivation *p* = 0.001; KD after sleep deprivation, *p* = 0.021; males KD no sleep deprivation, *p* = 0.025; KD after sleep deprivation, *p* = 0.007). In contrast, DI values in CD-fed rats did not differ significantly from chance (*p* > 0.05). Notably, CD males exposed to sleep deprivation showed a trend toward greater exploration of the familiar location (*p* = 0.069), suggesting a potential alteration in exploratory preference rather than full loss of spatial discrimination. Collectively, these results indicate that long-term KD enhances novelty-based spatial discrimination in rats of both sexes, maintaining performance above chance under baseline conditions and following sleep deprivation, while CD animals do not show a novelty preference and exhibit a slight shift toward familiar exploration after sleep deprivation.

### 3.7 The ketogenic diet decreases lipid but not protein oxidation in the hippocampus of aged female, but not male rats

**Figure 7:**
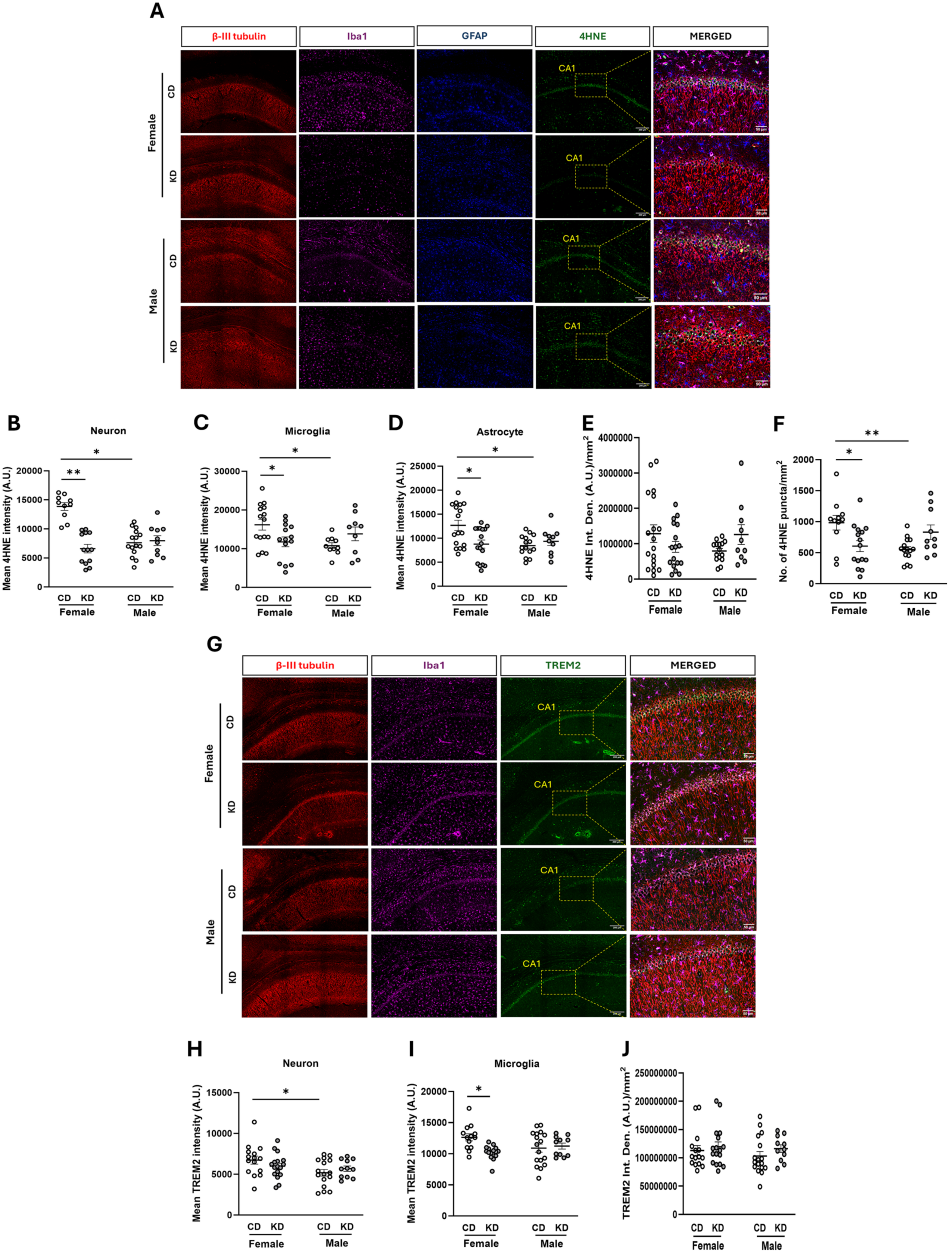
Effect of ketogenic diet intervention on oxidative stress and neuroinflammation in aged rats. **(A)** Representative immunofluorescence images showing *β*-III tubulin for neurons (red), Iba1 for microglia (cyan), GFAP for astrocytes (blue), and 4HNE (green) of the hippocampal region from coronal brain sections (50 μm) of aged male and female rats. The fifth column shows an enlarged merged image of the CA1 region of the hippocampus for the indicated groups. All images were acquired at 20X magnification and 3×2 tiles. Graphs showing the average intensity of 4HNE in neurons **(B)**, microglia **(C)**, and astrocytes **(D)**, respectively, quantified using the Ilastik-QuPath workflow for calculating the mean 4HNE intensity in each cell type expressed as arbitrary units (A.U.). Graphs showing the integrated density (Int. Den.) per mm^2^ expressed as arbitrary units (A.U.) **(E)** and no. of 4HNE puncta per mm^2^ **(F)** quantified using NIH-ImageJ software. **(G)** Representative immunofluorescence images showing β-III tubulin (red), Iba1 (cyan), and TREM2 (green) of the hippocampal region from coronal brain sections (50 μm) of aged male and female rats. The fourth column shows an enlarged merged image of the CA1 region of the hippocampus for the indicated groups. Graphs of mean TREM2 intensity in neurons **(H)** and microglia **(I)**, respectively quantified using the Ilastik-QuPath workflow for quantification of mean TREM2 intensity in each cell type expressed as A.U. **(J)** TREM2 integrated density per mm2 expressed as arbitrary units (A.U.) quantified using NIH-ImageJ software. Individual data points represent data from the CA1 region of the hippocampus from one brain tissue section; three brain sections were imaged per rat and approximately 100–200 cells were analyzed per cell type. The number of detections for each individual cell type for each marker is given in Supplementary Figures S3A–H, and the number of rats used for the analysis are given in Supplementary Table S1. Data represent Mean ± SEM. Mixed-effects two-way ANOVA (sex × diet) with animal as a random factor (fields nested within animals) and Tukey *post hoc* test. *p < 00.05, **p < 0.01. Control diet (CD) and ketogenic diet (KD).

Aging is associated with increased oxidative stress in the brain, driven in part by dysregulated mitochondrial function and diminished antioxidant capacity, which can contribute to neuronal injury and cognitive decline (Lin and Beal, 2006; Yin et al., 2016; Butterfield and Halliwell, 2019). To determine whether long-term KD reduces oxidative stress in an essential memory-related brain region, we examined markers of lipid and protein oxidation in the CA1 region of the hippocampus in rats. Hippocampal sections were immunolabeled for 4-HNE, a marker of lipid peroxidation, and 3-NT, a marker of protein oxidation. Cell-type–specific immunohistochemistry was performed to identify neurons (*β*-III tubulin, red), microglia (Iba1, cyan), and astrocytes (GFAP, blue) (Figure 7A). Quantification of 4-HNE intensity within each cell type was achieved using a machine learning–based workflow combining Ilastik and QuPath to generate probability maps for accurate cell detection, complemented by NIH ImageJ analysis of integrated density and puncta number. KD-fed female rats exhibited a significant reduction in 4-HNE immunoreactivity compared with CD females across all examined cell types, including neurons (*p* = 0.0026), microglia (*p* = 0.0349), and astrocytes (*p* = 0.0135) (Figures 7B–D). Statistical significance was assessed using a mixed-effects two-way ANOVA (sex × diet) with animal included as a random factor to account for nested sampling of fields within animals, followed by Tukey’s multiple comparisons test. We did not observe any significant differences between the groups in overall 4-HNE burden as measured by integrated density (Figures 7A,E). However, KD females showed a significant reduction in the number of 4-HNE punctas compared to CD females (*p* = 0.0181) using ImageJ-based analyses (Figures 7A,F). Notably, CD-fed females exhibited significantly higher levels of 4-HNE compared with CD-fed males across neurons (*p* = 0.0185), microglia (*p* = 0.0307), and astrocytes (*p* = 0.0101), indicating a sex difference in baseline lipid peroxidation within the hippocampus (Figures 7A–F). In contrast, male rats showed no significant differences in 4-HNE levels between KD and CD groups by either cell-type–specific or global measures. The number of cell detections included in this analysis has been given in Supplementary Figures S3A–C. In contrast to lipid oxidation, KD did not alter levels of the protein oxidation marker 3-NT in either females or males (Supplementary Figures S1A–E). The number of detected cells included in the 3-NT quantification analyses is shown in Supplementary Figures S3F–H. Together, these data demonstrate that KD selectively reduces lipid peroxidation but not protein oxidation, in hippocampal neurons, astrocytes, and microglia of aged female rats, with no detectable effect in males, indicating a sex-specific modulation of brain lipid redox homeostasis by ketogenic intervention.

### 3.8 The ketogenic diet selectively reduces microglial TREM2 in aged female rats without altering NLRP3 expression

Because neuroinflammation is a prominent feature of brain aging (“neuroinflammaging”), we next examined whether long-term KD modulates markers associated with inflammatory and microglial activation states, specifically TREM2 and the inflammasome component NLRP3. Although TREM2 has been reported to exert neuroprotective effects in some contexts (Raha et al., 2017; Wang et al., 2020), other studies indicate that reduced TREM2 signaling can attenuate neurodegeneration and associated neuroinflammatory responses (Leyns et al., 2017), highlighting the context-dependent nature of TREM2 regulation. To assess the effects of the KD, hippocampal sections were co-immunolabeled for neurons (β-III tubulin, red), microglia (Iba1, cyan), and TREM2 (green) (Figure 7G). TREM2 intensity within neuronal and microglial populations was quantified using the Ilastik–QuPath workflow described above. Neuronal TREM2 levels did not differ significantly between CD and KD groups in either females or males in response to KD. However, we found that CD females showed significantly higher neuronal TREM2 levels as compared to CD males (*p* = 0.0142; Figure 7H). In contrast, microglial TREM2 expression was significantly reduced in KD-fed female rats compared with CD females (*p* = 0.0167; Figure 7I), whereas no such change was observed in male rats. The number of cell detections included in these analyses is shown in Supplementary Figures S3D,E. Despite the cell-type–specific reduction in microglial TREM2 in KD females, overall hippocampal TREM2 intensity did not differ significantly between CD and KD groups in either sex (Figure 7J). We next assessed the effect of the KD on inflammasome-related signaling by quantifying NLRP3 (green) expression (Supplementary Figure S2A). No significant differences in NLRP3 expression were detected between CD and KD groups in neurons (Supplementary Figure S2B) or microglia (Supplementary Figure S2C) in either females or males. In astrocytes, baseline NLRP3 expression was significantly lower in males compared with females (*p* = 0.0130) and KD-fed females (*p* = 0.0166) (Supplementary Figure S2D); however, KD did not significantly alter astrocytic NLRP3 levels within either sex. Cell counts used for NLRP3 quantification are shown in Supplementary Figures S2F–H. There was no significant change in overall NLRP3 levels in KD versus CD within male or female groups, however, KD females showed significantly higher NLRP3 levels as compared to CD males (*p* = 0.0045; Supplementary Figure S2E). Together, these findings indicate that KD selectively reduced microglial TREM2 expression in female rats without altering neuronal TREM2 or NLRP3 expression, while males show no detectable changes in either marker, consistent with a sex-specific modulation of microglial state rather than global suppression of inflammatory signaling.

## 4 Discussion

In this study, we demonstrate that ketogenic interventions exert sex-dependent effects on sleep architecture, metabolic physiology, redox homeostasis, and cognitive resilience in aged rats. Importantly, these findings indicate that the neural impact of ketogenic interventions in aging reflects not only the presence of ketosis but also the metabolic and dietary context in which ketosis occurs, including biological sex, mitochondrial redox capacity, and diet composition. Collectively, these results identify REM sleep as a particularly metabolically sensitive phenotype that reveals sex-specific differences to ketone responsiveness in aging. Both exogenous ketone ester supplementation and long-term KD robustly elevated circulating ketone levels in males and females, but produced distinct downstream effects on glucose regulation, sleep–wake organization, and brain physiology. Acute KE exposure and chronic KD selectively enhanced REM sleep in aged females, via increased REM episode number, while producing no REM benefit, and in some cases opposing effects, in aged males. Despite these differences in sleep architecture, the KD supported novelty-based spatial discrimination in both sexes, maintaining performance above chance under baseline conditions and following sleep deprivation, whereas CD animals failed to show a consistent novelty preference and shifted toward familiar exploration. At the molecular level, KD selectively reduced lipid peroxidation in hippocampal neurons, astrocytes, and microglia of aged females without affecting protein oxidation, and was accompanied by a reduction in microglial TREM2 expression without changes in the inflammasome marker NLRP3. Together, these findings indicate that ketogenic interventions promote sex-specific adaptations in metabolic–sleep coupling and lipid redox regulation that support cognitive function in aging, with females showing a coordinated enhancement of REM sleep and lipid-associated microglial state.

### 4.1 Age-related differences in ketone utilization and brain function

Aging is associated with a progressive decline in cerebral glucose metabolism and mitochondrial efficiency, increasing vulnerability to energetic stress during states of high neuronal demand (Yin et al., 2014). In contrast, ketone uptake and oxidation remain relatively preserved in the aging brain (Cunnane et al., 2016; Goyal et al., 2017), providing a potential compensatory fuel source when glycolytic capacity is compromised. Ketone bodies such as BHB support ATP generation with a lower oxidative burden than glucose and additionally engage signaling pathways that regulate mitochondrial biogenesis, redox balance, and synaptic plasticity (Veech, 2004; Newman and Verdin, 2017). Consistent with these metabolic effects, KDs have been shown to modulate neural ensemble activity in frontal cortex and hippocampus in aged rats, indicating diet-induced metabolic shifts can influence large scale network dynamics (Hernandez et al., 2023).

Within this metabolic context, aging represents a state of energetic fragility in which the ability to utilize alternative substrates becomes increasingly consequential for brain function. The efficacy of ketone-based compensation, however, is not uniform and appears to be shaped by biological sex and hormonal milieu. Estrogen decline with aging has been linked to reduced mitochondrial efficiency, altered NAD^+^ metabolism, and diminished antioxidant capacity, potentially constraining ketone utilization in post-menopausal females or males with lower estrogenic tone (Yao and Brinton, 2012; Klinge, 2020). This framework provides a metabolic backdrop for understanding why ketogenic interventions may confer neural benefits in some aging contexts but not others, setting the stage for examining how specific brain states, particularly sleep, respond to improved energetic support.

### 4.2 Ketogenic modulation of REM sleep in aging

REM sleep is a metabolically active brain state in part due to sleep-dependent synaptic remodeling, memory consolidation, and cellular repair. Cortical glucose utilization and cerebral blood flow are comparable to wakefulness (Ramm and Frost, 1983; Maquet et al., 1990; Maquet et al., 1996; Nofzinger et al., 1997), yet occur in the absence of external sensory input and under conditions of markedly reduced monoaminergic neuromodulatory tone (Brown et al., 2012). This state of reduced neuromodulatory support during REM sleep, renders it relatively energetically inefficient and redox-vulnerable condition that is especially sensitive to mitochondrial dysfunction and oxidative stress. Accordingly, REM sleep integrity is one aspect of sleep architecture to deteriorate with aging and neurodegenerative disease (Mendelson and Bergmann, 1999; Wimmer et al., 2013). Age-related impairments in astrocyte–neuron metabolic coupling, mitochondrial biogenesis, and redox signaling likely contribute to this REM sleep instability (Belanger et al., 2011; Mander et al., 2017).

Within this framework, our finding that both acute KE supplementation (Figures 2A,C) and chronic KD (Figure 3A) selectively enhanced REM sleep in aged female rats, but not aged male, suggests that ketone availability interacts with sex-specific metabolic and redox biology to lower the energetic barriers to REM sleep expression in late life. Notably, KD increased the number of REM episodes (Figure 3B) without altering duration (Figure 3C) or EEG spectral power (Figure 4C), indicating enhanced REM sleep initiation rather than changes in REM microarchitecture. By contrast, aged males failed to exhibit REM enhancement (Figure 3A) despite equivalent elevations in circulating ketones, indicating that substrate availability alone is insufficient to restore REM sleep without accompanying improvements in mitochondrial efficiency or redox buffering capacity.

Rather than implying a direct causal role for REM sleep in mediating cognitive outcomes, these data support a model in which REM sleep functions as a sensitive indicator of neural metabolic resilience. The enhancement of REM sleep in females coincided with reduced hippocampal lipid peroxidation (Figures 7A–F) and selective modulation of microglial lipid-associated signaling, suggesting that improved lipid redox homeostasis may enable the brain to sustain energetically demanding REM states without incurring oxidative damage. In this view, REM sleep reflects the capacity of aging neural circuits to support metabolically costly internal activity under conditions of improved energy efficiency, rather than acting as a singular mechanistic driver of memory performance.

### 4.3 Sex differences in ketone metabolism and redox responsiveness

Aged females and males differed markedly in their metabolic and redox responses to ketogenic interventions, consistent with extensive evidence for sexual dimorphism in metabolic flexibility and mitochondrial regulation. Females generally exhibit a greater capacity to shift toward lipid- and ketone-based metabolism during energetic stress (Cameron-Smith et al., 2003; Mauvais-Jarvis, 2015). Estrogen promotes *β*-oxidation and ketone utilization through activation of PPARα and AMPK signaling while enhancing antioxidant defenses and limiting lipid peroxidation. Further, estrogen deficiency accelerates senescence-associated immune activation and inflammation in adipose tissue (Imano et al., 2023). Consistent with this framework, KDs have been shown to preferentially improve mitochondrial function and cognition in female rodents (Pathak et al., 2022), and to induce more favorable redox and inflammatory profiles in females compared with males (Smolensky et al., 2023). In contrast, male metabolic responses to lipid-rich diets appear more vulnerable to oxidative imbalance. A study using a hydrogenated Crisco–based KD, rich in polyunsaturated, oxidatively unstable fats, induced MnSOD acetylation, 4-hydroxynonenal accumulation, and p53-dependent senescence exclusively in males (Wei et al., 2024; Wei et al., 2025). These effects were mitigated by estrogen supplementation or antioxidant treatment, implicating estrogen-dependent regulation of mitochondrial detoxification pathways as a key determinant of sex-specific outcomes. Our findings extend this work by demonstrating that even when ketosis is achieved using oxidatively stable fat sources, aged males fail to exhibit the REM sleep enhancements (Figure 3A) observed in females, despite showing preserved cognitive discrimination (Figure 6). This pattern suggests that loss of estrogenic support with aging constrains certain aspects of ketone responsiveness in male brains. In addition to estrogen signaling, sex-linked differences in mitochondrial DNA regulation, lipid handling, and immune–metabolic crosstalk may further contribute to differential ketone responsiveness in aging.

### 4.4 Sex hormones effect on sleep wake regulation and quality in aging

Metabolic sex differences intersect closely with sleep regulation, where neuronal excitability, redox state, and circadian timing converge. Estrogen and progesterone modulate neuronal excitability, adenosine signaling, and GABAergic tone in sleep-regulatory nuclei such as the preoptic area, hypothalamus, and brainstem (Lancel et al., 1996; Dorsey et al., 2020). These hormones also enhance mitochondrial respiration and antioxidant enzyme expression (Simpkins et al., 2010; Yao and Brinton, 2012; Klinge, 2020), conferring resilience during energetically demanding states like REM sleep. Our data show that aged females under ketogenic conditions exhibited enhanced REM sleep, consistent with redox-driven stabilization of neural networks.

In addition to REM-specific effects, sleep homeostasis is critically indexed by slow-wave activity, particularly delta power during NREM sleep, which reflects the accumulation and dissipation of sleep pressure (Borbely and Achermann, 1999). Following sleep deprivation, increased NREM delta power is typically interpreted as a marker of elevated homeostatic sleep drive and greater recovery need. In our study, aged CD-fed females exhibited delta rebound following sleep deprivation; however, KD-fed females did not have a significant increase in delta power over the 6 h recovery period but showed a trend toward more rapid normalization of delta power compared with CD-fed females (Figures 5B,C). This attenuated delta rebound in KD females suggests a reduced homeostatic sleep recovery requirement rather than impaired sleep homeostasis, consistent with improved metabolic efficiency and reduced neuronal stress during prior wakefulness. Our spectral findings therefore support a model in which ketogenic intervention in aged females not only enhances REM sleep (Figures 2C, 3A) expression but also reduces the intensity of compensatory NREM recovery (Figures 5B,C), indicating improved sleep–wake efficiency. Rather than reflecting impaired sleep homeostasis, lower delta power during recovery sleep in KD females likely reflects reduced accumulation of sleep pressure, potentially due to enhanced mitochondrial function, improved redox balance, and more efficient energy utilization during wake and REM sleep. This interpretation is consistent with evidence that metabolic state and mitochondrial function strongly influence sleep pressure dynamics and slow-wave generation.

Theta oscillations play distinct roles across vigilance states. During NREM sleep, theta activity is not a defining feature and is often associated with lighter sleep or state instability. Both male and female KD-fed rats exhibited reduced theta power during NREM sleep (Figure 4B), consistent with stabilization of NREM architecture and reduced intrusion of wake- or REM-like activity. This reduction in theta was accompanied by a decrease in total EEG power during NREM sleep, suggesting a shift toward more efficient and less diffuse oscillatory activity rather than impaired sleep depth. Importantly, this pattern occurred without evidence of increased sleep pressure, as KD-fed females did not show an exaggerated delta rebound during recovery sleep. Theta oscillations during REM sleep are a core feature of hippocampal–cortical coordination and are closely linked to REM sleep intensity and functional quality. The preservation of REM theta power in KD-fed females (Figure 4C), alongside increased REM episode number (Figure 3B), suggests maintenance of REM network integrity. In contrast, the reduction of REM theta power in males (Figure 4C) may indicate weaker engagement of REM-related oscillatory circuits, consistent with the absence of REM sleep enhancement and limited metabolic benefit in this group.

In contrast, aged KD males, lacking estrogenic protection did not show comparable improvements in REM sleep or changes in delta power dynamics (Figures 5B,C), likely reflecting impaired coupling between metabolic state and electrophysiological regulation of sleep. These results parallel human studies showing that women maintain greater REM integrity and recover more efficiently from sleep loss (Carrier et al., 2001), and that sex differences in lipid metabolism influence physiological responses to dietary interventions (D'Abbondanza et al., 2020; Aronica et al., 2021). Together, these findings support a model in which estrogen acts as both a metabolic amplifier and electrophysiological stabilizer of ketone benefits.

In females, estrogen maintains mitochondrial integrity through SIRT3-dependent deacetylation of MnSOD, limits lipid peroxidation, and promotes ketone-driven ATP production, enabling optimal synaptic maintenance, efficient sleep homeostasis, and sleep-dependent memory consolidation (Ayala et al., 2010; Flor et al., 2017; Pathak et al., 2022; Smolensky et al., 2023; Kumar et al., 2024). In males, the absence of estrogenic regulation likely leads to heightened vulnerability to oxidative stress and lipid peroxidation, particularly under diets containing unstable fats. Thus, sex differences in ketone metabolism and sleep physiology reflect not only divergent REM sleep regulation but also fundamental differences in sleep homeostatic control, shaped by mitochondrial redox capacity and hormonal status.

### 4.5 Behavioral expression of cognitive resilience and exploratory strategy in aging

Although aging is associated with fragmented sleep and reduced sleep quality, aged animals still exhibit homeostatic responses to additional sleep loss (McKillop et al., 2018; Campos-Beltran and Marshall, 2021). Acute sleep deprivation is therefore commonly used as an experimental perturbation to probe sleep homeostasis and neural resilience in aging models. In the present study, this approach allowed us to test whether KD intervention improves the ability of aged animals to respond to an additional sleep challenge beyond the baseline sleep alterations associated with aging. Sex-dependent differences in metabolic and sleep resilience were reflected in exploratory behavior under baseline conditions and following sleep deprivation. Importantly, reduced novelty preference in object- and place-based tasks is now recognized to reflect shifts in exploratory strategy rather than memory failure per se. Classic and recent work has emphasized that the critical indicator of intact memory in these assays is the ability to discriminate between novel and familiar stimuli, whereas the direction and magnitude of exploratory preference can be modulated by factors such as age, stress such as sleep deprivation, arousal, and motivational state (Ennaceur and Delacour, 1988; Antunes and Biala, 2012). Under this framework, failure to discriminate, reflected by equal exploration of novel and familiar locations, provides stronger evidence of memory impairment than reduced novelty preference alone (Swiercz et al., 2025). Aged CD rats did not consistently discriminate objects from chance (Figure 6). This interpretation is consistent with prior reports showing that aged Fischer-344 rats exhibit diminished novelty preference (Maasberg et al., 2012). In contrast, KD–fed rats had robust novelty-oriented exploration and discrimination performance above chance (Figure 6), indicating preservation of both spatial discrimination and adaptive exploratory drive. Taken together, these behavioral findings suggest that ketogenic intervention enhances resilience to cognitive stressors in aging by stabilizing exploratory behavior under challenging conditions, rather than simply increasing memory capacity. When considered alongside preserved REM sleep and improved lipid redox balance in females, these results support a broader model in which metabolic flexibility sustains adaptive cognitive function in late life.

### 4.6 Mechanistic integration: ketone-driven redox modulation and sleep

To our knowledge, this is the first demonstration that ketogenic intervention selectively reduces hippocampal lipid peroxidation and modulates microglial lipid-associated signaling in a sex-dependent manner in aged animals (Figure 7). The divergent outcomes of ketogenic interventions across studies underscore that ketosis alone is insufficient to predict neural benefit consistent with the broader metabolic context highlighted below. Rather, diet composition, lipid oxidative stability, and endogenous redox buffering capacity critically shape metabolic and neural outcomes (Ayala et al., 2014; Wei et al., 2025). Diets enriched in oxidatively unstable fats can impose a substantial lipid peroxidation burden that overwhelms mitochondrial defenses, particularly in males (Wei et al., 2024; Wei et al., 2025). In contrast, our molecular data indicate that a cocoa butter–based KD diet exert selective, rather than global, effects on brain stress pathways. Specifically, the KD reduced lipid peroxidation in aged females but not males, while markers of protein oxidation remained unchanged. Lipid peroxidation products such as 4-hydroxynonenal are among the most sensitive indicators of oxidative stress and can vary independently of protein oxidation, particularly in membrane-rich tissues such as brain (Esterbauer et al., 1991; Ayala et al., 2014). This pattern suggests that ketogenic intervention preferentially improves lipid redox homeostasis, potentially by reducing membrane-associated oxidative burden or enhancing lipid-specific antioxidant defenses, rather than broadly suppressing oxidative damage (Sullivan et al., 2004; Veech, 2004; Maalouf et al., 2009).

Aging is characterized by a complex neuroinflammatory milieu (“inflammaging”) driven by multiple interacting cellular and metabolic processes, including mitochondrial dysfunction, oxidative stress, and altered immune signaling (Yin et al., 2014). The KD did not alter the inflammasome marker NLRP3 (Supplementary Figure S2) but reduced TREM2 in aged females. Because TREM2 is closely linked to lipid sensing, cholesterol metabolism, and microglial state transitions, reduced TREM2 in the context of decreased lipid peroxidation may reflect diminished lipid-driven microglial activation or a reduced need for a compensatory lipid-handling program (Wang et al., 2015; Ulland et al., 2017; Nugent et al., 2020). However, given TREM2’s additional roles in microglial survival and phagocytic function, the functional implications of this change should be interpreted cautiously and will require validation using complementary markers of microglial activation and function (Krasemann et al., 2017; Gratuze et al., 2023). Together, these findings support a model in which ketone metabolism preferentially limits lipid peroxidation and modulates microglial lipid-handling without directly suppressing inflammasome signaling. The absence of changes in NLRP3 despite reduced oxidative stress is consistent with prior work demonstrating that microglial metabolic and lipid-handling programs can be regulated independently of canonical inflammasome activation (Heneka et al., 2013; Keren-Shaul et al., 2017; Deczkowska et al., 2018). Such early redox-dependent mechanisms may be particularly relevant for preserving sleep and cognition, as REM sleep is highly energy-demanding and exquisitely sensitive to mitochondrial and redox perturbations (Cirelli and Tononi, 2008; Wimmer et al., 2013; Mong and Cusmano, 2016). REM sleep may therefore serve as a sensitive functional biomarker of metabolic resilience during aging.

Importantly, several features of the diet used in the present study were designed to minimize confounding variables that can influence metabolic and cognitive outcomes. First, the CD and KDs contained equivalent protein content, reducing the possibility that differences in protein availability or protein restriction contributed to the observed physiological effects. Second, the KD did not significantly alter body weight in our aged animals. This is notable because KDs can variably induce weight loss or weight gain depending on macronutrient composition, caloric density, and experimental context (Goldberg et al., 2020; Lu et al., 2023). The absence of body weight differences suggests that the sleep, behavioral, and molecular effects observed here are more likely attributable to ketogenic metabolism rather than secondary consequences of altered body mass. Diet composition and lipid source are also important determinants of KD outcomes. In the present study, the KD was formulated using cocoa butter as the primary fat source, which is composed predominantly of saturated and monounsaturated fatty acids that are relatively resistant to lipid peroxidation. In contrast, some studies reporting adverse effects of KDs have used fat sources such as hydrogenated vegetable shortening (Crisco) (Wei et al., 2025), which contains higher levels of oxidation-prone polyunsaturated fatty acids and can increase lipid peroxidation and inflammatory signaling (Ayala et al., 2014). These dietary factors may interact with age-related changes in brain lipid biology. Brain lipid composition is known to shift with aging, including alterations in phospholipid composition, fatty acid saturation, and membrane lipid signaling pathways that influence mitochondrial function and susceptibility to oxidative stress (Soderberg et al., 1991; Yin et al., 2014; Naudi et al., 2015). Sleep disruption has also been shown to alter lipid metabolism and membrane phospholipid turnover in the brain (Weljie et al., 2015; Niazi et al., 2025). Because KDs substantially alter systemic lipid availability and fatty acid metabolism, dietary lipid composition may interact with these age- and sleep-related lipid remodeling processes. Thus, the physiological effects of ketogenic interventions likely reflect not only ketone metabolism itself but also diet-dependent differences in lipid stability, membrane remodeling, and redox balance within the broader context of age-related neuroinflammation.

### 4.7 Implications and translational relevance

These findings have major implications for designing safe and effective ketogenic therapies for aging, sleep, and neurodegenerative disorders. Together with previous studies, our results indicate that the success or failure of ketogenic interventions depends on fat quality, redox buffering capacity, and hormonal milieu, not simply the level of ketosis achieved. Adverse outcomes associated with oxidatively unstable fats emphasize the need for “redox-optimized ketosis” strategies that: (1) Prioritize lipid stability, favoring monounsaturated and medium-chain triglycerides (medium chain triglycerides, olive oil, coconut oil) over oxidatively unstable fats. (2) Incorporate antioxidant reinforcement, such as N-acetylcysteine, coenzyme Q10, or manganese superoxide dismutase mimetics, particularly in males and post-menopausal individuals. (3) Personalize by sex and hormonal status, recognizing estrogen’s central role in mediating mitochondrial and electrophysiological benefits of ketosis. Such precision metabolic approaches may enhance sleep quality, reduce oxidative stress, and improve cognitive resilience in older adults at risk for neurodegenerative disease.

### 4.8 Limitations and future directions

Several limitations should be considered. First, our experiments were conducted exclusively in aged rats (22–25 months), a period characterized by substantial variability in physical stamina, sensory acuity, and motivational drive. While this model is well suited for studying late-life metabolic fragility, it limits our ability to determine how responsiveness to ketogenic interventions evolves across the lifespan. Future studies incorporating young and middle-aged cohorts will be necessary to define age- and stage-specific windows of metabolic and sleep plasticity. Second, participation in the novel place preference task was reduced in a subset (19%) of aged animals. Decreased exploration and task engagement are common in very old rodents and likely reflect age-related changes in motivation, locomotor capacity, or stress responsivity rather than cognitive impairment per se. This limited behavioral assessment in some animals despite intact electrophysiological recordings. While the novel place preference task is advantageous in minimizing training demands, its reliance on spontaneous exploration poses inherent challenges in advanced aging. Future studies will incorporate complementary cognitive assays that involve motivated behaviors to increase participation. Third, although ketogenic interventions reduced lipid oxidative stress and TREM2, we did not observe changes in overall inflammatory indices (NLRP3). This dissociation suggests that ketone-mediated benefits may operate upstream of overt neuroinflammatory activation, particularly in aged brains. Further markers of systemic inflammation will be important for defining the inflammatory landscape associated with metabolic and sleep interventions. Fourth, while our findings implicate mitochondrial redox regulation as a key mediator of ketone effects on REM sleep and cognition, we did not directly measure mitochondrial acetylation status, NAD^+^/NADH balance, or sirtuin activity in sleep-regulatory regions. Direct interrogation of these pathways particularly within the preoptic area or brainstem arousal control nuclei will be essential to establish causal links between ketone metabolism, redox signaling, and sleep–wake regulation. Finally, sex differences emerged as a central feature of our findings; however, we did not manipulate hormonal status directly. The contribution of estrogen signaling to ketone responsiveness from our study remains inferential. Wei et al. (2025) suggests a causal role of estrogen but future studies incorporating ovariectomy, hormone replacement, or pharmacologic modulation of estrogen receptors will be critical to confirm the proposed model in which estrogen acts as a permissive factor for ketone-mediated neural resilience.

## 5 Conclusion

Together, these findings support a unified model in which ketone metabolism promotes metabolic resilience in the aging brain, enabling the expression of energetically demanding REM sleep and preserving adaptive cognitive behavior through redox-mediated stabilization of mitochondrial function, with estrogen acting as a key permissive factor. By integrating electrophysiological, behavioral, and molecular measures, this study positions REM sleep as a sensitive indicator of neural metabolic sufficiency rather than a singular mechanistic driver of cognitive outcomes. These results highlight biological sex and redox state as critical determinants of responsiveness to ketogenic interventions and underscore the importance of optimizing both nutrient composition and biological context when translating metabolic therapies to aging and neurodegenerative disease.

## Data Availability

The original contributions presented in the study are included in the article/Supplementary material, further inquiries can be directed to the corresponding author.
