# Supplementary material for: Ketogenic interventions enhance REM sleep in females and support memory in aged rats
Source: Front Aging Neurosci. 2026 Apr 10;18:1797686. doi: 10.3389/fnagi.2026.1797686 (PMC13106476; doi:10.3389/fnagi.2026.1797686)
Supplement: Supplementary file 1 [file Data_Sheet_1.pdf]

Supplementary Material

Description	Number of rats
Old female rats (Control)	6
Old female rats (Ketogenic diet)	8
Old male rats (Control)	5-6
Old male rats (Ketogenic diet)	3-4

Supplementary Table 1: The total number of rats used for immunohistochemical analysis.

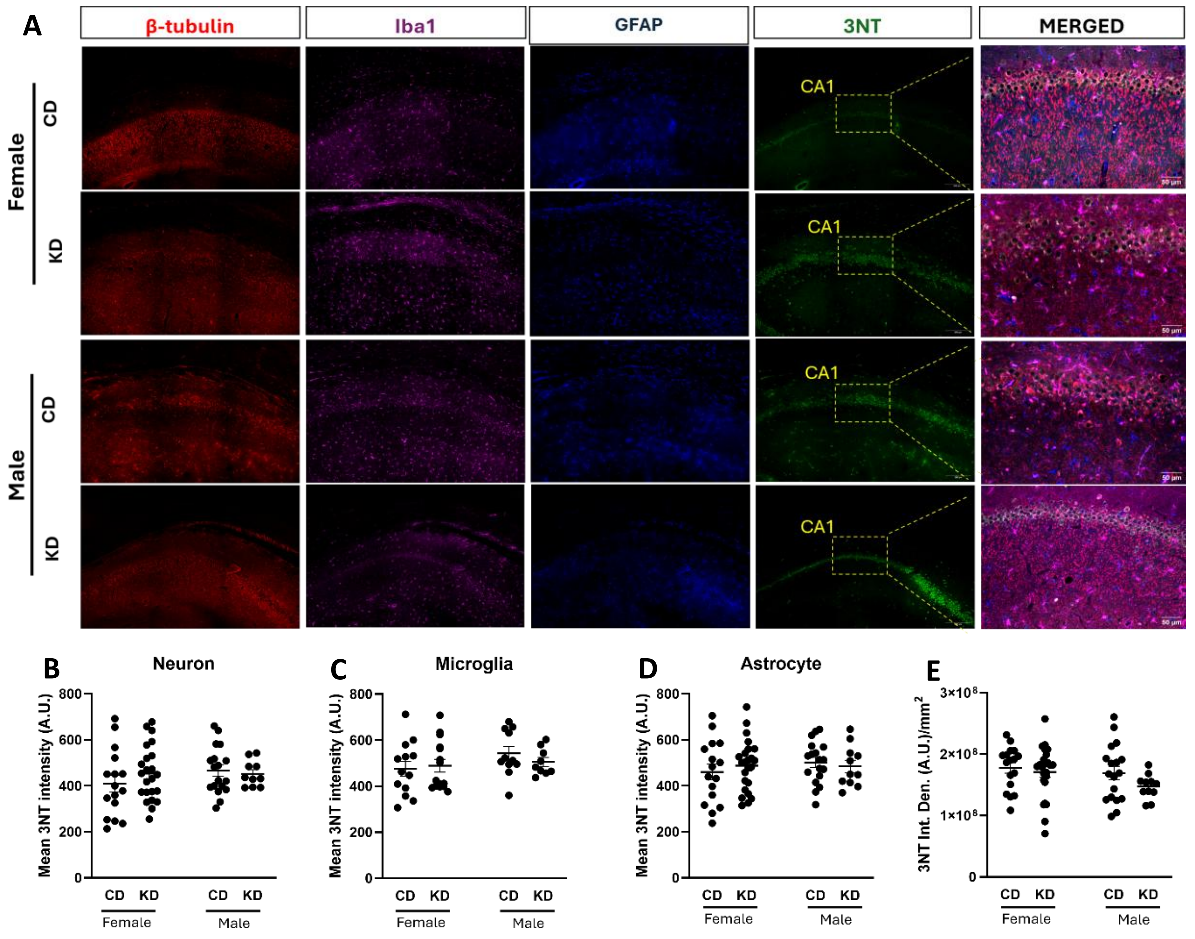

Supplementary Figure 1: Effect of ketogenic diet intervention on oxidative stress in aged rats. (A) Representative immunofluorescence images showing β-III-tubulin (red), Iba1 (purple), GFAP (blue) and 3NT (green) of the hippocampal region from the coronal brain sections (50 µm) of aged rats. Graph of mean 3NT intensity in neurons (B), microglia (C) and astrocytes (D), respectively quantified using the Ilastik-QuPath workflow for quantification of mean 3NT intensity in each cell type expressed as A.U. Graph showing the integrated density (Int. Den.) per mm2 (E), quantified using the NIH-ImageJ software. Individual data points represent each field of view from one brain tissue section, three brain sections were imaged per rat, approximately 100-200 cells were analyzed per image by batch analysis. The number of rats used for the study are given in Table S1. Data represent Mean±SEM. Mixed-effects two-way ANOVA (sex × diet) with animal as a random factor (fields nested within animals) and Tukey post hoc test. There were no significant main effects or sex by diet interactions. $^{*}p<0.05$, $^{**}p<0.01$. control diet (CD) and ketogenic diet (KD)

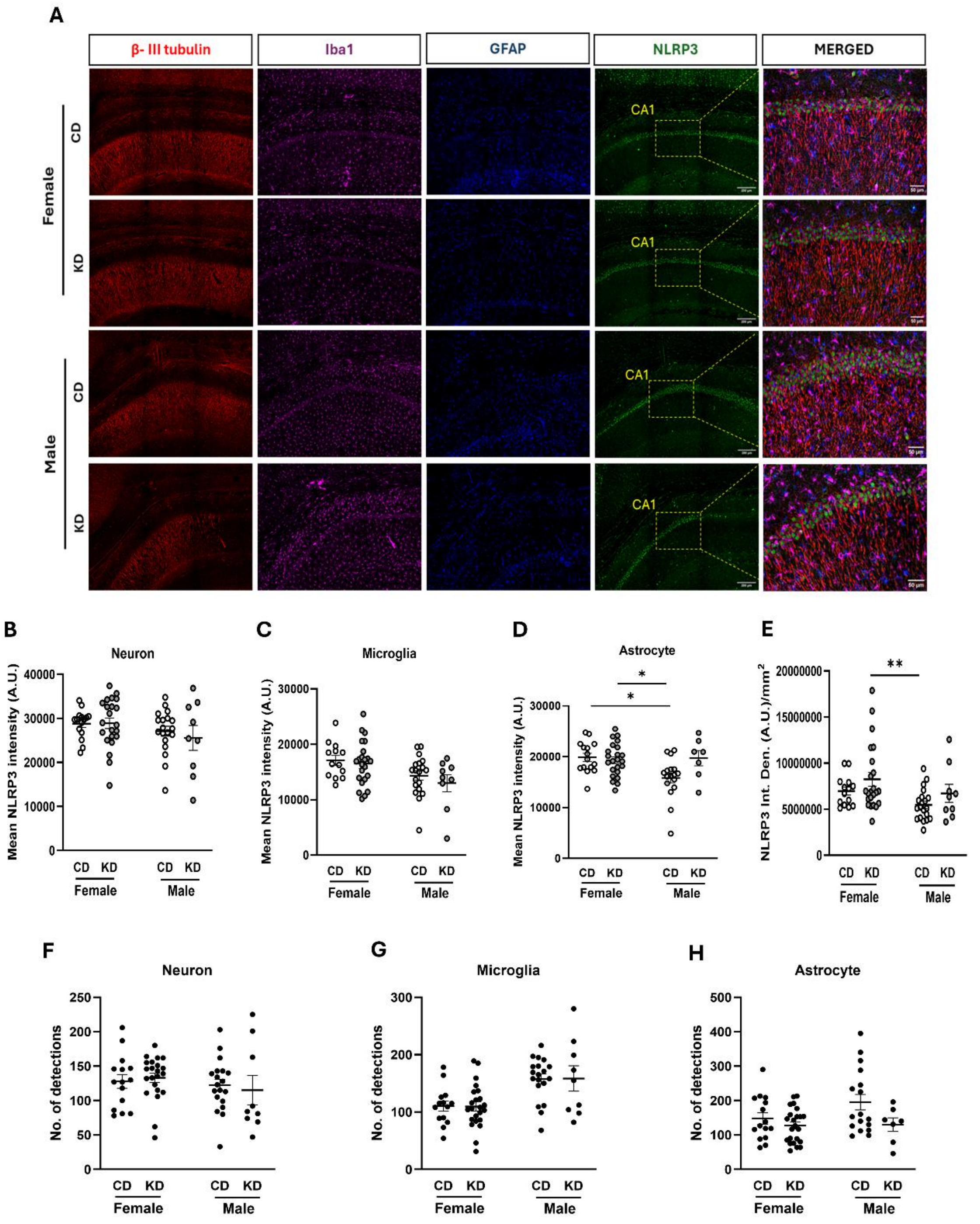

Supplementary Figure 2: Effect of ketogenic diet intervention on neuroinflammation in aged rats. (A) Representative immunofluorescence images showing β-tubulin (red), Iba1 (purple), GFAP (blue) and NLRP3 (green) of the hippocampal region from the coronal brain sections (50 µm) of aged male and female rats. The fifth column shows an enlarged merged image of the CA1 region of the hippocampus for the indicated groups. Graph showing the average intensity of NLRP3 in neurons (B), microglia (C) and astrocytes (D), respectively, quantified using the hybrid Ilastik-QuPath workflow for calculation of mean NLRP3 intensity in each cell type expressed as arbitrary units (A.U.). Graphs showing the integrated density (Int. Den.) per mm^2 (E), quantified using the NIH-ImageJ software. Graphs showing the number of cell detections for neurons (F), microglia (G) and astrocytes (H) quantified using Ilastik-QuPath workflow. Individual data points represent each field of view from one brain tissue section, three brain sections were imaged per rat, approximately 100-200 cells were analyzed per image by batch analysis. The number of rats used for the study are given in Table S1. Data represent Mean±SEM. Mixed-effects two-way ANOVA (sex × diet) with animal as a random factor (fields nested within animals) and Tukey post hoc test. *$p<0.05$, **$p<0.01$. control diet (CD) and ketogenic diet (KD)

Number of detections for each cell type- 4HNE

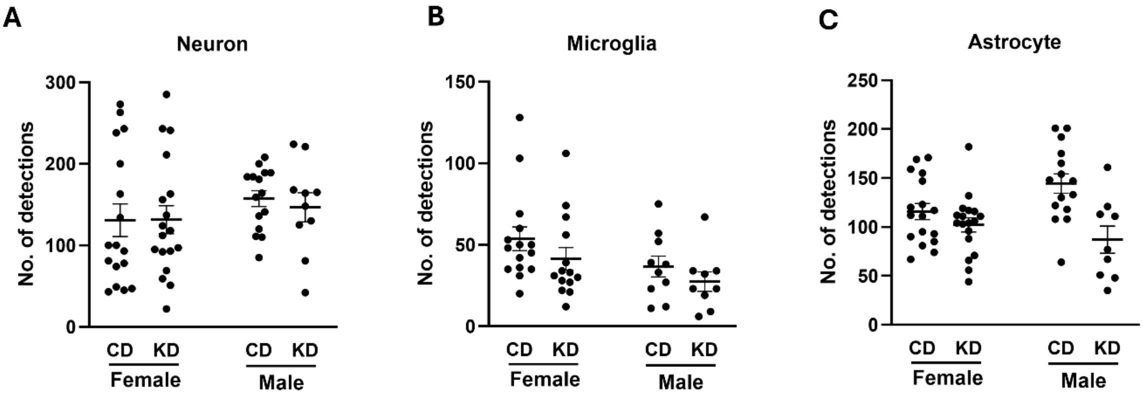

Number of detections for each cell type- TREM2

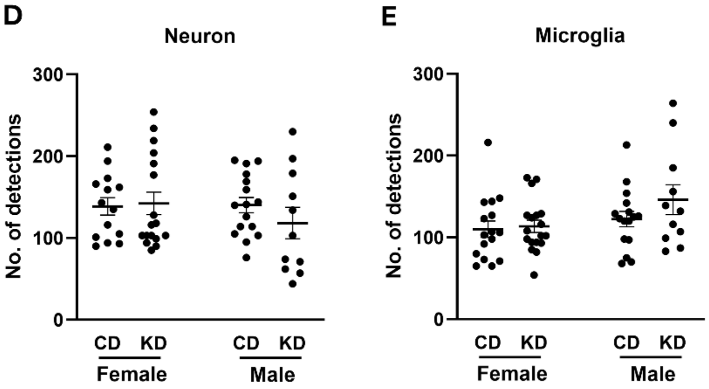

Number of detections for each cell type - 3NT

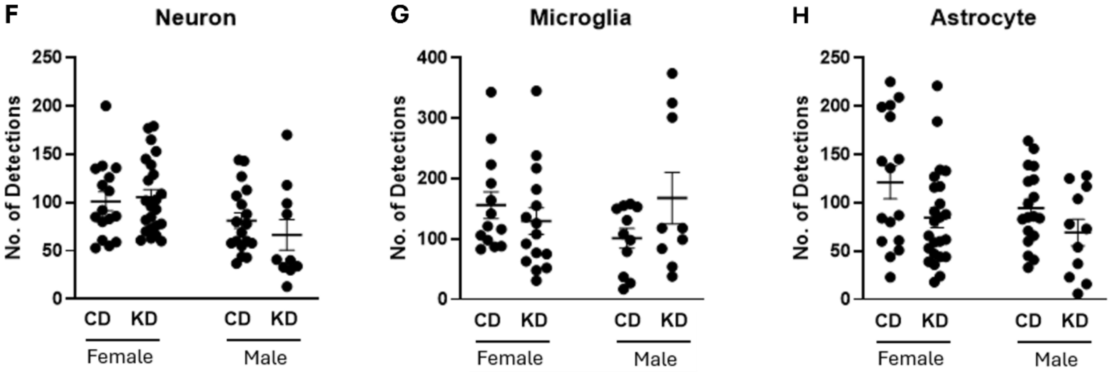

Supplementary Figure 3: Number of cell detections of each individual cell type quantified using the hybrid Ilastik-QuPath method. Graphs showing the number of neurons (A), microglia (B) and astrocytes (C) used for mean intensity calculation of 4HNE. Graphs showing number of neurons (D) and microglia (E) for calculation of mean intensity of TREM2. Graphs showing the number of detections of neurons (F), microglia (G) and astrocytes (H) for calculation of mean intensity of 3NT. Individual data points represent number of each cell type (neuron, microglia or astrocyte as indicated) for the groups as indicated for each field of view. Three brain slices were imaged per rat, number of rats used for the analysis is given in Table S1. control diet (CD) and ketogenic diet (KD)
